# New Insights into the Structural Requirements of Isatin-Derived Pro-Apoptotic Agents against Acute Myeloid Leukemia

**DOI:** 10.3390/ph15121579

**Published:** 2022-12-17

**Authors:** Ahmed K. Hamdy, Takashi Sakamoto, Tsugumasa Toma, Masaharu Sakamoto, Mohammed A. S. Abourehab, Masami Otsuka, Mikako Fujita, Hiroshi Tateishi, Mohamed O. Radwan

**Affiliations:** 1 Medicinal and Biological Chemistry Science Farm Joint Research Laboratory, Faculty of Life Sciences, Kumamoto University, Kumamoto 862-0973, Japan; 2 Department of Medicinal Chemistry, Faculty of Pharmacy, Assiut University, Assiut 71526, Egypt; 3 Department of Pharmaceutics, College of Pharmacy, Umm Al-Qura University, Makkah 21955, Saudi Arabia; 4 Department of Drug Discovery, Science Farm, Ltd., Kumamoto 862-0976, Japan; 5 Chemistry of Natural Compounds Department, Pharmaceutical and Drug Industries Research Institute, National Research Centre, Giza 12622, Egypt

**Keywords:** isatin, indolin-2-one, acute myeloid leukemia, apoptosis, ERK1/2, MAPK

## Abstract

Searching for bioactive compounds within the huge chemical space is like trying to find a needle in a haystack. Isatin is a unique natural compound which is endowed with different bio-pertinent activities, especially in cancer therapy. Herein, we envisaged that adopting a hybrid strategy of isatin and α,β-unsaturated ketone would afford new chemical entities with strong chemotherapeutic potential. Of interest, compounds **5b** and **5g** demonstrated significant antiproliferative activities against different cancer genotypes according to NCI-60 screening. Concomitantly, their IC_50_ against HL-60 cells were 0.38 ± 0.08 and 0.57 ± 0.05 µM, respectively, demonstrating remarkable apoptosis and moderate cell cycle arrest at G1 phase. Intriguingly, an impressive safety profile for **5b** was reflected by a 37.2 times selectivity against HL-60 over PBMC from a healthy donor. This provoked us to further explore their mechanism of action by in vitro and in silico tools. Conclusively, **5b** and **5g** stand out as strong chemotherapeutic agents that hold clinical promise against acute myeloid leukemia.

## 1. Introduction

Cancer remains a global public health consideration which causes mortality and brings great pressure to human lives all over the world [1]. As a result of the diagnosis of cancer being in advanced stages in most patients, chemotherapy is still the primary treatment for cancer over surgical operations. Despite the huge effort made by researchers for the development of new drugs for the eradication of cancers, there was no radical cure [2]. There is an urgent need for development of new chemotherapeutic drugs with good efficacy, potency, selectivity, and low toxicity. Among various types of cancers, acute myeloid leukemia (AML) was responsible for 20,050 cases and 11,540 deaths in the United States in 2022, according to the American Cancer Society’s estimates. AML is one of the most common types of leukemia in adults, but also it occurs in children as well [3,4]. AML starts in the bone marrow white blood cells (other than lymphocytes), then it quickly moves into the blood as well. Sometimes, it can spread to other organs and tissues inside the body such as the liver, spleen, lymph nodes, central nervous system, and testicles [5,6,7]. AML is responsible for a lack of normal hematopoietic cells, resulting in severe symptoms such as anemia, dyspnea, bleeding and threatening infections due to poorly differentiated progenitor cells [8]. 

**Figure 1:**
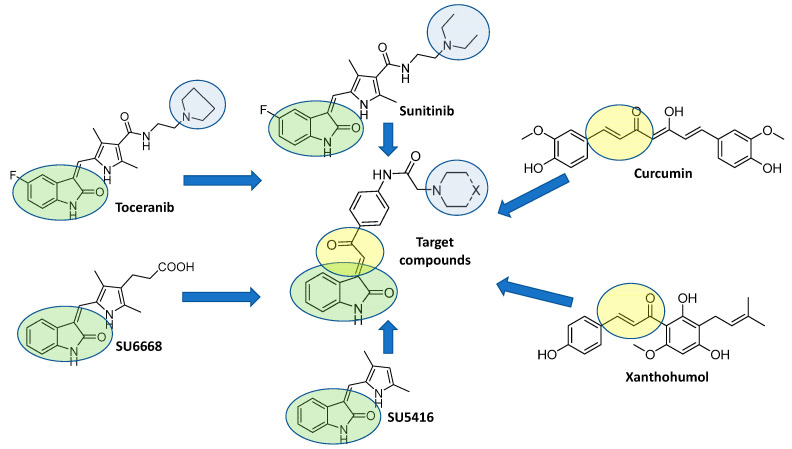
Rational design of the target compounds based on oxindole hybridization with α, β-unsaturated ketone.

Isatin (2,3-dioxindole) is a privileged scaffold in the field of medicinal chemistry and drug discovery due to its broad range of biological actions, as well as its wide liability to chemical modifications. Consequently, many researchers from academia and industry sought to develop isatin-based anticancer drugs during the last few decades [9,10,11]. Fortunately, these great efforts were accomplished with the discovery of new clinically approved anticancer drugs such as sunitinib, toceranib and nintedanib. Moreover, varieties of compounds containing isatin scaffold revealed exceptional anti-cancer activities in clinical trials, including semaxanib (SU5416) and orantinib (SU6668) [12,13]. Isatin analogues were found to be effective against numerous tumors including AML [14,15,16]. Isatin-based derivatives exert their anticancer activity through modulation of different biological targets such as tyrosine kinase, histone deacetylase, tubulin, and extracellular signal-regulated protein kinase (ERK1/2) phosphorylation, resulting in apoptosis and, moreover, influencing apoptosis-related gene expression [17,18,19,20] (Figure 1).

The α,β-unsaturated ketone (Michael acceptor) scaffold has been considered as a common structural feature in many pharmacologically active natural products and synthetic compounds including calichemicin, prostaglandin A2, brefeldin A, pikromycin, ceftibuten, asparenomycin and epoxyquinomycin [21,22]. In addition to, the naturally occurring chemopreventive agent curcumin, which displays strong anti-cancer activity [23]. Moreover, chalcones, other typical edible natural products, also have a wide range of pharmacological activities, such as xanthohumol. α,β-unsaturated ketone is crucial for the biological activities of curcumin and chalcones, as its omission abolishes their activities [24,25,26,27]. Furthermore, it was reported that the incorporation of α,β-unsaturated ketone into molecules often resulted in drugs with enhanced cytotoxicity. Hence, α,β-unsaturated ketone can be regarded as a functionality structure for drug design [28,29].

To date, molecular hybrids are the one of most prevalent strategies to develop new anti-cancer hit compounds based on combining the structural features of two different active scaffolds, which not only decrease the adverse effects but also enhance the biological activities [30,31]. In this study, inspired by the structural features of isatin-derived approved drugs and α,β-unsaturated ketone, we proposed that combining these bioactive structural features together in a single molecule may result in the development of promising new lead compounds with potential anticancer activity (Figure 1). Therefore, owing to our continuous concerns regarding design, synthesis and biological evaluation of anticancer activity [32,33,34,35,36,37], we adopted a molecular hybridization strategy to design a novel series of oxindole-based analogues bearing α,β-unsaturated ketone to produce potential anticancer agents.

## 2. Rational Design

It was reported that oxindole scaffold is crucial for anticancer activity of isatin derivatives as it can occupy the hinge region of the ATP binding domain in numerous kinases [38]. In addition, the hydrophobic tail such as *N*,*N*-diethyl in sunitinib is responsible for binding to the allosteric site of various kinases [39]. Therefore, in this study, the oxindole scaffold and the hydrophobic tail were retained and linked through α,β-unsaturated ketone spacer. We tried different bio-isosteric modifications in the hydrophobic tail, such as extension of the flexible *N*,*N*-diethyl moiety of sunitinib to *N*,*N*-dipropyl **5b**, structure rigidification using piperidine **5c** or morpholine **5d**. Moreover, extension to *N*-alkyl **5e**, *N*-acyl **5f** and **5g**, or *N*-heteroaryl piperazine **5h** were examined.

## 3. Results and Discussions

### 3.1. Chemistry

**Scheme 1:**
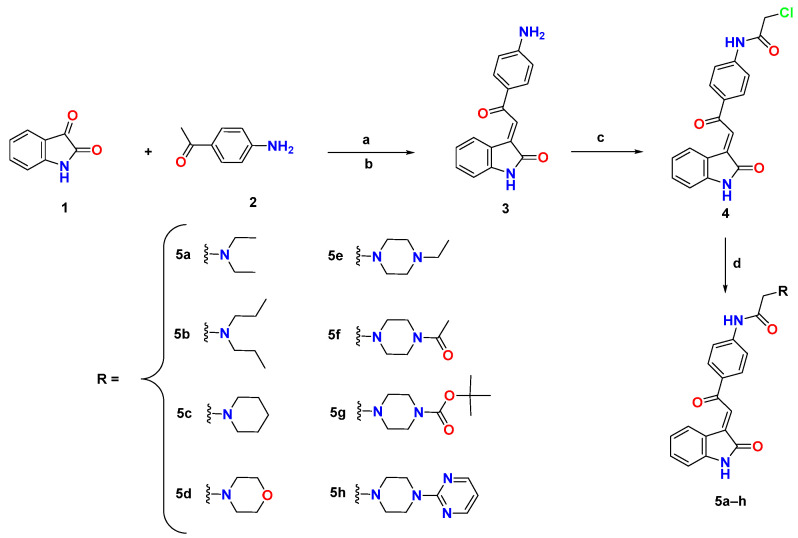
Synthesis of the target compounds **5a**–**h**. Reagent and conditions: (a) EtOH, diethylamine, r.t., overnight; (b) EtOH, hydrochloric acid, reflux, 3 h; (c) Chloroacetyl chloride, DIPEA, THF, 0 °C, 20 min. then r.t., 3 h; (d) Secondary amine, potassium iodide, DMF, r.t., 3 h.

The target compounds were synthesized as depicted in Scheme 1, through aldol condensation of isatin **1** with *p*-aminoacetophenone **2** in ethanol, in the presence of diethylamine. The white precipitate formed was filtered and subjected to a dehydration reaction through refluxing with hydrochloric acid in ethanol to give the intermediate **3**. The free amino group in compound **3** was reacted with chloroacetyl chloride in super dehydrated tetrahydrofuran to afford the chloroacetamide intermediate **4**. Then, we synthesized the final target compounds through stirring of **4** with different aliphatic and alicyclic secondary amines in super-dehydrated DMF, in the presence of a catalytic amount of potassium iodide. All the final compounds were obtained in good yields after purification using flash column chromatography.

### 3.2. Biological Evaluation

#### 3.2.1. Evaluation of the In Vitro Cytotoxic Activity

To assess cytotoxic activity of the synthesized compounds against several cancer cell lines which represent different body organs and tissues, we submitted all compounds to National Cancer Institute (NCI), Bethesda, within the Developmental Therapeutics Program (DTP). All compounds were selected by NCI for in vitro screening of anticancer activity according to the protocol of the Drug Evaluation Branch of the National Cancer Institute. Primary in vitro one-dose anticancer assay was performed in full NCI-60 cell lines at a single concentration (10 µM) against 60 cell lines derived from nine tumor subpanels, including leukemia, melanoma, lung, colon, CNS, ovarian, renal, prostate and breast cancer cell lines [40]. The in vitro growth inhibition and lethality were determined and depicted in Table 1 as percentages as follows: growth inhibition (GI%) (values between 0 and 100) and lethality (values less than 0), for example a value of −40 would mean 40% lethality. Additionally, the results of NCI single dose assay were illustrated as bars in the single-dose mean graphs (Supplementary Materials, Figures S21–S28).

Overall, NCI-60 screening revealed that the synthesized compounds possess strong cytotoxic activity against several cancer cell lines. All compounds showed exceptional activity against leukemia cell lines, especially acute myeloid (AML) leukemia cells HL-60 and acute lymphoblastic leukemia (ALL) cells MOLT-4. For example, compound **5b** and **5g** showed 43.91% and 46.44% lethality against HL-60, and 31.26% and 44.85% lethality against Molt-4 cells. Furthermore, all target compounds revealed prominent activity against colon cancer cell lines, especially human colorectal cancer cell lines HCT116 and SW-620. Treatment with compound **5b** resulted in lethality of 88.15% and 76.08% of HCT116 and SW-620, respectively, and in the same way, compound **5g** exerted strong lethality on HCT116 and SW-620 cells (96.98% and 88.72%, respectively). Regarding cells derived from non-small cell lung cancer, HOP-92, NCI-H226 and NCI-H22 were the most affected cell lines while the tested compounds showed weak growth inhibition against A549/ATCC with GI% values ranging from 5.75% to 25.07%. Within CNS cancer cell lines, glioblastoma cells SF-539 were the most affected line, where **5a** showed an inhibition in growth of 58.59%. Moreover, compounds **5b** and **5g** induced lethality of 35.29% and 72.33% in SF-539, respectively. In addition, the synthesized compounds manifested spectacular activity against some melanoma cancer cell lines, especially LOX IMVI cells. Where, compound **5h** inhibit cells growth by 59.67%, while compounds **5a**, **5b** and **5g** induced lethality of LOX IMVI cells by 61.45%, 98.98 and 100%, respectively. The synthesized compounds exhibited good to excellent activities against all renal cancer cell lines except A498. Likewise, the compounds affected growth of prostate cancer cell line P53 with GI% between 4.42% and 74.73%. Finally, all synthesized compounds revealed fabulous cytotoxic activity against breast cancer cell lines especially MCF-7. Additionally, the compounds exerted outstanding activity against triple negative breast cancer cell line MDA-MB-486 with GI% ranging from 49.41% to 76.47%. Furthermore, **5g** induced 67.01% lethality. 

**Figure 2:**
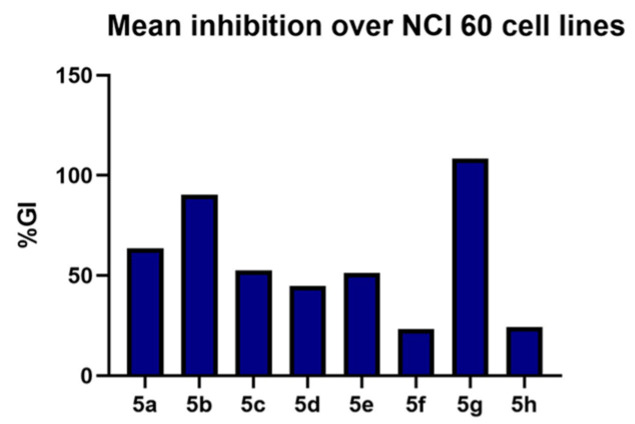
Graphical representation of the mean inhibition percentages calculated according to GI% values over NCI-60 cell lines.

Based on the NCI screening data, we can conclude that the present hybrid strategy is a fruitful approach to obtain novel compounds with remarkable antiproliferative activity against different cancer types. Extension of the acyclic hydrophobic tail *N*,*N*-diethyl **5a** to the longer *N*,*N*-dipropyl **5b** highly enhanced the antiproliferative activity where the mean GI% was 90.45. On the contrary, substituent rigidification using piperidine **5c** or morpholine **5d** led to a trivial drop in the activity with mean GI% values of 52.52 and 44.76, respectively. In a similar vein, substitution with *N*-ethyl piperazine **5e** afforded slightly less potent compounds such as **5c** and **5d**. While changing the ethyl group of compound **5e** into acetyl group as in **5f** resulted in a dramatic activity decline with a mean GI% 23.22, using a *t*-butyloxycarbonyl substitution (**5g**) highly escalated the activity with a mean value of 8.44% lethality. However, using pyrimidine substituted piperazine (**5h)** lowered the activity to a similar value of **5f**. In short, compounds **5b** and **5g** are the most active compounds within the series (Figure 2).

Inspired by these interesting NCI cytotoxicity results, we tried to measure the IC_50_ of synthesized compounds against proliferation of acute myeloid leukemia HL-60 cells, as it was one of the most sensitive cell lines in the one-dose screening. The highly potent kinase inhibitor staurosporine was used as a reference in this assay. Upon treatment of cells with different doses from the synthesized compounds, it revealed impressive cytotoxicity with sub-micromolar IC_50_ values for compounds **5a**, **5b**, **5g** and **5h**, as shown in Table 2. Compounds **5b** and **5g** were the most potent with IC_50_ values 0.38 ± 0.08 µM and 0.57 ± 0.05 µM, which are comparable with the positive control staurosporine. To assess the safety of the synthesized compounds, we screened the cytotoxicity of the most potent compound **5b** on the normal peripheral blood mononuclear cells (PBMC). The compound showed weak cytotoxicity on normal cells with an IC_50_ value of 14.17 µM, and selectivity index (SI) of 37.2. These results demonstrated that the synthesized compounds have selective cytotoxic activity against cancer cells, without affecting normal body cells. Therefore, the target compounds are promising anticancer lead compounds.

#### 3.2.2. Analysis of Apoptosis/Necrosis Induction by **5b** and **5g**

**Figure 3:**
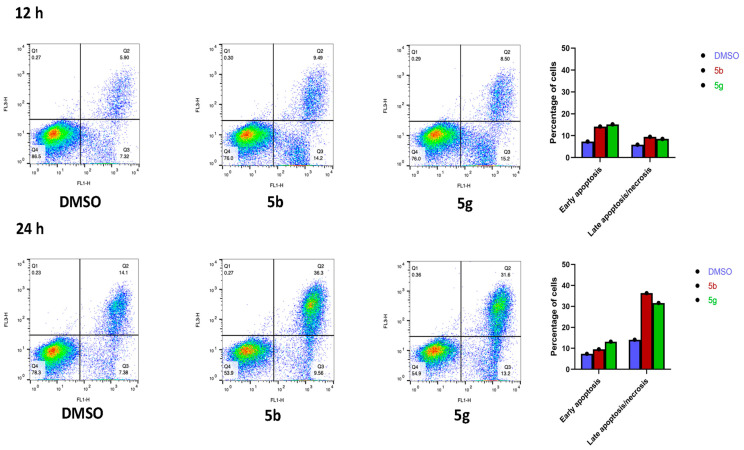
Apoptosis analysis of **5b** and **5g** using Annexin V-FITC (FL1-H)/7-AAD (FL3-H) dual staining in HL-60 cells.

Annexin V-based flow cytometry analysis is used to identify the mechanism of cell death, whether achieved through programmed apoptosis or non-specific necrosis [41]. Therefore, Annexin V-FTIC/7-AAD dual staining assay was undertaken for the most active compounds **5b** and **5g** on HL-60 cells. Both compounds induced early apoptosis by approximately double that of DMSO after 12 h incubation. Moreover, both compounds showed a two-fold increase in the percentage of late apoptosis/necrosis after 24 h incubation compared to DMSO. These data confirm that the target compounds affect cancer cells mainly through the induction of apoptosis (Figure 3).

#### 3.2.3. Cell Cycle Analysis

**Figure 4:**
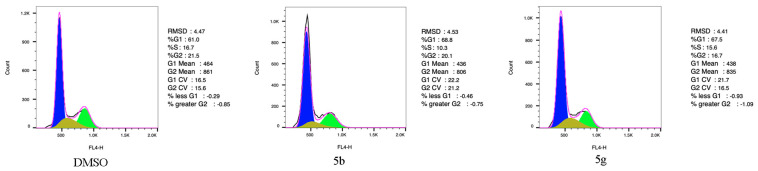
Cell cycle analysis of the HL60 cells treated with compound **5b** and **5g** for 24 h using flow cytometry.

The effect of the most active compounds **5b** and **5g** on cell cycle distribution was assessed using flow cytometric analysis in HL-60 cells. Upon treatment of cells with compounds **5b** and **5g** for 24 h, both compounds showed an increase in the percentage of cells in the G1 phase compared with the control, as depicted in Table 3 and Figure 4. These data revealed that the target compounds have the tendency to arrest the cell cycle in the G1 phase (Figure 4).

#### 3.2.4. Western Blot Analysis

To explore the mechanism of action of the synthesized compounds and the most probable target inside the cells, we used the Swiss target prediction tool [42]. Swiss target prediction tool showed that ERK1/2 are among the most probable targets of the synthesized compounds. The extracellular signal-related kinases 1 and 2 (ERK1/2) are serine/threonine kinases that play a key role in signal transduction of growth factors. ERK1/2 are the terminal kinases in the RAS-RAF-MEK-ERK signaling cascade (MAPK pathway), which is crucial for signal transduction from cell surface receptors to transcription factors inside the nucleus and regulation of gene expression inside cells. Stimulation of receptor tyrosine kinase (RTK) by mitogens, growth factors and cytokines recruit and activate RAS, which in turn activates the Raf family (A-Raf, B-Raf and C-Raf). Active Raf phosphorylates and activates the dual-specificity kinases, MEK1 and its homologue MEK2 (MEK1/2), which sequentially phosphorylate the serine/threonine kinases ERK1/2 [43]. Phosphorylated ERK1/2 (P-ERK1/2) catalyze and phosphorylate 250 cytosolic and nuclear downstream substrates including transcription factors (e.g., Ets-1, Elk-1, c-Fos, c-Jun), and the RSK family of kinases, phosphatases, apoptotic proteins, and cytoskeletal proteins, among many others. This wide array of substrates regulates many cellular functions, such as gene transcription, cell cycle progression, proliferation, migration, adhesion, survival and metabolism [44]. Furthermore, the importance of ERK1/2 for the transition of cells from G1 to S phase in the cell cycle, and its ability to inhibit cell apoptosis through inhibition and phosphorylation of caspase 9 was reported [45].

In a particular relevance to oncology, mutations in RAS or RAF result in activation of the RAS-RAF-MEK-ERK signaling cascade (MAPK pathway) in over 30% of cancers including melanoma, lung, pancreatic, colorectal (CRC) cancer and acute myeloid leukemia (AML) [46,47]. There are approved drugs on the market which act through inhibition of BRAF and MEK1/2 kinases, however their therapeutic benefits are limited by resistance resulting from BRAF amplification or MEK mutation which reinstate ERK activity. Presence of ERK1/2 in the distal end of the RAS-RAF-MEK-ERK pathway, and because it is rarely undergone mutations, make ERK1/2 an attractive target for the development of anticancer drugs. Currently, the design and synthesis of compounds with antitumor activity through targeting ERK1/2 have increased and some of them entered clinical trials, but no drug has been approved to date [43,48]. 

**Figure 5:**
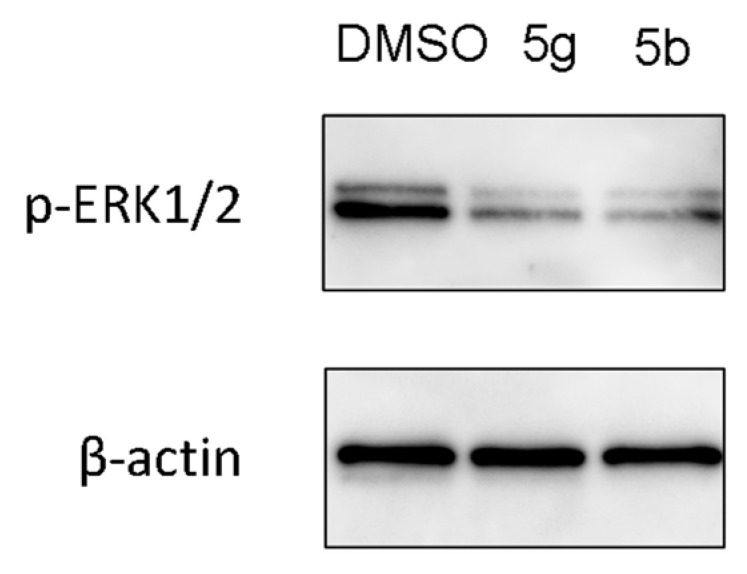
Effect of compounds **5b** and **5g** on the immunoblotting of p-ERK1/2 proteins.

Based on the results of molecular target prediction using the Swiss target prediction tool, we measured the effect of the most active compounds **5b** and **5g** on phosphorylation of ERK1/2 in HL-60 cells. Where, HL-60 cells were incubated with compounds **5b** and **5g** for 24 h, then levels of phosphorylated ERK1/2 (p-ERK1/2) were evaluated using Western blot analysis. The results, depicted in Figure 5, explore how the target compounds significantly blocked phosphorylation of ERK1/2 by about 60% and 80% for compounds **5b** and **5g,** respectively. The effect of compounds **5b** and **5g** on ERK1/2 phosphorylation seems to be responsible for their cytotoxic activity, induction of apoptosis and cell cycle arrest in the G1 phase. 

## 4. In Silico Screening Studies

### 4.1. Molecular Docking

**Figure 6:**
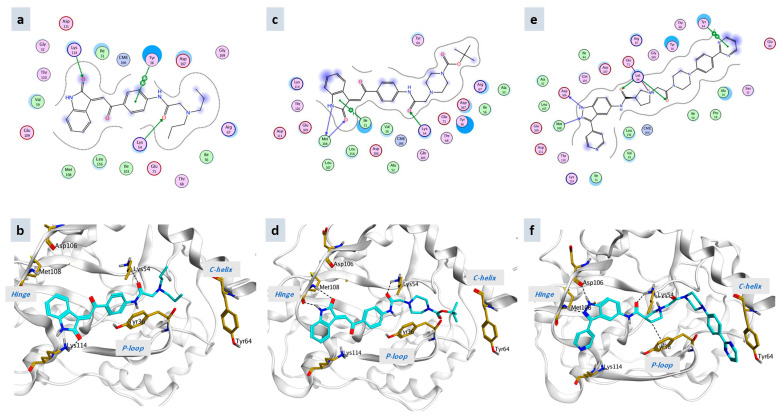
(**a**) 2D representation of the ligand interactions and (**b**) 3D representation of the docking of **5b** in the active site of ERK2 (PDB ID: 6GDM); (**c**) 2D representation of the ligand interactions and (**d**) 3D representation of the docking of **5g** in the active site of ERK2 (PDB ID: 6GDM); (**e**) 2D representation of the ligand interactions and (**f**) 3D representation of the docking of SCH772984 in the active site of ERK2 (PDB ID: 6GDM).

The majority of ERK inhibitors undergoing clinical trials are ATP competitive, blocking ERK1/2 catalytic activity without affecting their phosphorylation by MEK or translocation of p-ERK to the nucleus. SCH772984 is a selective potent ERK1/2 inhibitor which can inhibit both catalytic activity and phosphorylation of ERK1/2 by MEK. Therefore, SCH772984 can suppress the MAPK pathway signaling completely and overcome emergence of resistance. Crystal structure of ERK2 co-crystallized with SCH772984 revealed that it binds to altered conformation of ERK2 in which Tyr36 in the glycine-rich loop (P-loop) becomes tucked under the loop as depicted in Figure 6. Binding of SCH772984 is hypothesized to be responsible for its ability to block ERK1/2 phosphorylation [40,48].

In order to validate the obtained activity of the synthesized compounds on the level of ERK phosphorylation, the synthesized compounds were virtually screened within the binding site of SCH772984 with ERK2. The crystal structure of ERK2 co-crystallized with SCH772984 (PDB ID: 6GDM) which was downloaded from the protein data bank [47,49]. Docking of the most active compounds (**5b** & **5g**) and redocking of the native ligand (SCH772984) were performed inside the reported binding site of SCH772984 using commercial software Molecular Operating Environment (MOE 2019). The docking results analysis of the 2D and 3D poses, shown in Figure 6, illustrated that they were well-fitted on the ERK2 binding site and exhibited optimistic interactions with the key amino acid residues. The most active compounds **5b** and **5g** showed binding to the key amino acids inside the ATP hinge and the glycine rich loop (P-loop). Compound **5b** interacted at the hinge region through hydrogen binding with Lys114, it also showed hydrogen binding with Lys54 and pi–pi interaction with Tyr36 at the p-loop. On the other hand, compound **5g** revealed hydrogen binding with Met108 at the hinge and Lys54 at the p-loop. Therefore, compounds **5b** and **5g** showed the same binding theme as SCH772984. Moreover, compounds **5b** and **5g** have good binding energies scores (–8.8002 Kcal/mol and −8.6110 Kcal/mol respectively) which are comparable with the binding energy score of SCH772984 (−9.8950 Kcal/mol). The docking study results suggested that the synthesized compounds bind to ERK and block its phosphorylation and these data are in accordance with the Western blot results.

### 4.2. In Silico Prediction of Physicochemical and Pharmacokinetic Properties

Appropriate pharmacokinetic properties are very significant for efficacy and safety in drug development. Many potent compounds fail to become drugs due to their inappropriate pharmacokinetics. In the current study, the SwissADME website (http://www.swissadme.ch/index.php accessed on 18 November 2022) was utilized for prediction of the physicochemical and pharmacokinetic properties of the synthesized compounds **5a**–**h** in comparison to sunitinib. Gastrointestinal absorption is a critical pharmacokinetic behavior for a drug to be taken orally. As the oral route is the most convenient for most patients, the estimation of gastrointestinal absorption is crucial in the drug discovery processes.

The Brain Or IntestinaL EstimateD permeation method (BOILED-Egg) is an accurate predictive model for both gastrointestinal absorption and brain permeation. BOILED Egg is a plot of polarity expressed in TPSA versus lipophilicity expressed in WLOGP. The white region of BOILED Egg plot represents the physicochemical space of compounds with highest probability of being passively absorbed by the gastrointestinal tract, while the yellow region (yolk) represents the physicochemical space of compounds with highest opportunity for BBB permeability. Another model for rapid estimation of drug-likeness is the bioavailability radar which is a plot of six different physicochemical properties namely, size (SIZE), polarity (POLAR), lipophilicity (LIPO), solubility (INSOLU), flexibility (FLEX) and saturation (INSATU). The central pink hexagon region illustrates the optimal range for each property (SIZE: MW between 150 and 500 g/mol, lipophilicity: XLOGP3 between −0.7 and +5.0, polarity: TPSA between 20 and 130Å^2^, solubility: log S not higher than 6, saturation: fraction of carbons in the sp^3^ hybridization not less than 0.25, and flexibility: no more than 9 rotatable bonds [50,51].

**Figure 7:**
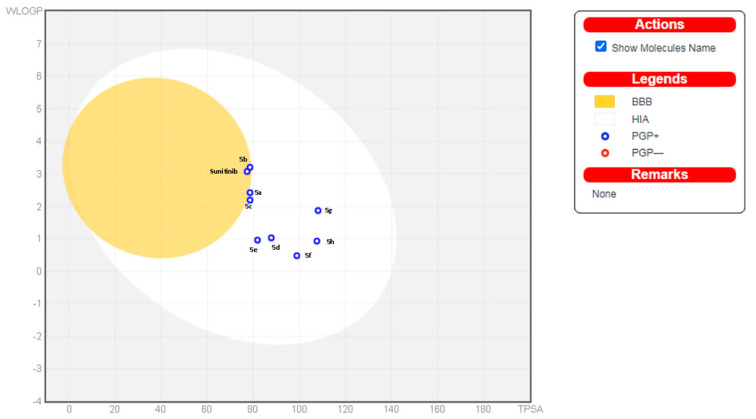
BOILED-Egg depiction of the synthesized compounds and sunitinib.

**Figure 8:**
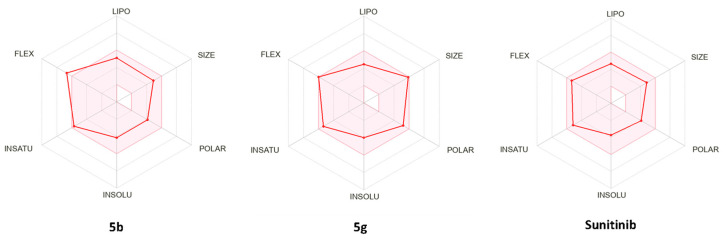
Bioavailability radar of compounds **5b**,**5g** versus sunitinib.

Based on these calculations, target compounds **5a**–**h** revealed favorable parameters as compared to sunitinib, which are presented in Table 4. As depicted in Table 4, BOILED egg model Figure 7 and bioavailability radar Figure 8, all target compounds were predicted to have high GI absorption similar to sunitinib. All target compounds obeyed the Lipinski rule without violation and showed reasonable polarity (TPSA 78.51–108.05 Å^2^) and credible molecular masses (below 500 g/mol). Only compound **5b** is predicted to pass through the BBB but it is predicted to be effluated outside of the CNS by P-glycoprotein. Therefore, the synthesized compounds are promising anticancer lead compounds with favorable pharmacokinetic properties.

## 5. Conclusions

In the present study, we adopted a hybrid strategy for isatin and α,β-unsaturated ketone to afford new hybrids with eminent antiproliferative effects. The hitherto unreported compounds were purified and identified by different spectroscopic tools. Compounds **5b** and **5g** demonstrated the highest activity through the NCI-60 screening. Their IC_50_ values against HL-60 cells were 0.38 ± 0.08 and 0.57 ± 0.05 µM, respectively. Both compounds triggered unequivocal apoptosis and cell cycle arrest at the G1 phase, presumably by interaction with ERK2 and inhibition of ERK1/2 phosphorylation. Furthermore, **5b** showed 37.2 times selectivity towards HL-60 over PBMC. The titled compounds possess favourable physicochemical and pharmacokinetic properties making them promising chemotherapeutic agents that warrant further optimization and development.

## 6. Materials and Methods

### 6.1. Chemistry

The chemicals were purchased from Sigma-Aldrich (St. Louis, MO, USA), Kanto Chemical (Tokyo, Japan), Nacalai Tesque (Kyoto, Japan), Tokyo Chemical Industry (Tokyo, Japan), and FUJIFILM Wako (Osaka, Japan). The commercially available reagent-grade chemicals were used without further purification. The reaction progress was monitored by thin-layer chromatography (TLC) on TLC silica gel 60 F254 aluminum sheets (Merck, Darmstadt, Germany). The flash column chromatography was performed on Silica Gel 60N (40–100 mesh, Kanto Chemical). ^1^H-NMR and ^13^C-NMR spectra were recorded on a Bruker Avance 600 spectrometer (600 MHz) (Billerica, MA, USA). Mass spectrometry (MS) and high-resolution mass spectrometry (HRMS) data were recorded on a JEOL (Tokyo, Japan) JEOL JMS-700M Station using a positive fast atom bombardment (FAB) technique with 3-nitrobenzyl alcohol as the matrix.

#### 6.1.1. Synthesis of (E)-3-(2-(4-Aminophenyl)-2-oxoethylidene)indolin-2-one (**3**)

To a solution of **1** (3 g, 10 mmol, 1 equiv.) in ethanol (20 mL), equivalent amounts of *p*-aminoacetophenone **2** (2.75 g, 10 mmol, 1 equiv.) and diethylamine (2 mL) were added. The mixture was stirred at room temperature overnight. The white precipitate formed was filtered and washed with ethanol and water. The obtained crude product was dissolved in ethanol (30 mL), conc. HCl (1 mL) was added, then the mixture was refluxed for 2 h. The mixture was cooled and poured into ice water. The reddish precipitate formed was collected by filtration and washed with water. The crude product was purified using column chromatography using hexane/ethyl acetate (1:1) as the mobile phase.

Yield: 83%; reddish powder; ^1^H-NMR (600 MHz, DMSO/*d*_6_) δ 10.71 (s, 1H), 7.88 (d, J = 7.6 Hz, 1H), 7.81–7.77 (m, 2H), 7.61 (s, 1H), 7.29 (td, J = 7.7, 1.2 Hz, 1H), 6.91 (td, J = 7.6, 1.0 Hz, 1H), 6.88–6.84 (m, 1H), 6.65–6.61 (m, 2H), 6.39 (s, 2H). ^13^C-NMR (150 MHz, DMSO/*d*_6_) δ 187.97, 168.37, 154.79, 144.17, 134.08, 131.87, 131.36, 128.17, 126.35, 124.70, 121.53, 120.26, 112.95, 110.10.

#### 6.1.2. Synthesis of (E)-2-Chloro-N-(4-(2-(2-oxoindolin-3-ylidene)acetyl)phenyl)acetamide (**4**)

Chloroacetyl chloride (0.85 g, 7.5 mmol, 1 equiv.) was added dropwise to a solution of **3** (2 g, 7.5 mmol, 1 equiv.) and DIPEA (2 mL, 11.25 mmol, 1.5 equiv.) in tetrahydrofurane (30 mL) at 0 °C. The mixture was stirred for 20 min at 0 °C and at room temperature for a further 3 h. The reaction mixture was poured into ice water and the reddish precipitated formed was collected by filtration. The crude product was washed with hydrochloric acid (1M), then water. The crude product was purified by column chromatography using n-hexane/ethyl acetate (1:1) as the mobile phase.

Yield: 72%; reddish powder; ^1^H-NMR (600 MHz, DMSO/*d*_6_) δ 10.79 (s, 1H), 10.74 (s, 1H), 8.14–8.05 (m, 2H), 8.11–8.08 (m, 2H), 7.99 (d, J = 7.7 Hz, 1H), 7.99 (d, J = 7.7 Hz, 1H), 7.88–7.77 (m, 2H), 7.83–7.80 (m, 2H), 7.71 (s, 1H), 7.71 (s, 1H), 7.34 (td, J = 7.7, 1.2 Hz, 1H), 7.34 (td, J = 7.7, 1.2 Hz, 1H), 6.95 (td, J = 7.7, 1.0 Hz, 1H), 6.95 (td, J = 7.7, 1.0 Hz, 1H), 6.91–6.87 (m, 1H), 6.90–6.88 (m, 1H), 4.33 (s, 2H). ^13^C-NMR (150 MHz, DMSO/*d*_6_) δ 189.77, 168.19, 165.32, 144.77, 143.59, 135.98, 132.70, 132.29, 130.13, 126.63, 126.22, 121.69, 119.99, 119.02, 110.31, 43.58. MS (FAB) *m*/*z* 378.3 (M+H)^+^; HRMS (FAB) calcd. for C_18_H_14_N_2_O_3_Cl: 341.0693, found: 341.0707.

#### 6.1.3. General Procedure for Synthesis of **5a**–**h**

Compound **4** (100 mg, 0.29 mmol, 1 equiv.) with few crystals of potassium iodide was dissolved in DMF (2 mL). The corresponding secondary amine (3.0 equiv.) was added dropwise. The reaction mixture was stirred at room temperature for about 3 h. After the reaction monitored by TLC was over, brine was added to the reaction mixture under stirring, and the suspended mixture was filtered and washed with water. The crude residue was purified by column chromatography on silica gel to obtain the final products.

#### 6.1.4. (E)-2-(Diethylamino)-N-(4-(2-(2-oxoindolin-3-ylidene)acetyl)phenyl)acetamide (**5a**)

Mobile phase dichlromethane/methanol (99:1). Yield: 61%; reddish powder; ^1^H-NMR (600 MHz, DMSO/*d*_6_) δ 10.78 (s, 1H), 10.06 (s, 1H), 8.08–8.04 (m, 2H), 7.97 (d, *J* = 7.8 Hz, 1H), 7.91–7.86 (m, 2H), 7.70 (s, 1H), 7.33 (td, *J* = 7.7, 1.2 Hz, 1H), 6.94 (td, *J* = 7.7, 1.0 Hz, 1H), 6.88 (d, *J* = 7.7 Hz, 1H), 3.22 (s, 2H), 2.61 (q, *J* = 7.1 Hz, 4H), 1.02 (t, *J* = 7.1 Hz, 6H). ^13^C-NMR (150 MHz, DMSO/*d*_6_) δ 189.79, 170.75, 168.22, 144.71, 143.62, 135.85, 132.66, 131.91, 130.00, 126.58, 126.41, 121.70, 119.99, 118.98, 110.32, 57.38, 47.75, 11.88. MS (FAB) *m*/*z* 378.3 (M+H)^+^; HRMS (FAB) calcd. for C_22_H_24_N_3_O_3_: 378.1818, found: 378.1809.

#### 6.1.5. (E)-2-(Dipropylamino)-N-(4-(2-(2-oxoindolin-3-ylidene)acetyl)phenyl)acetamide (**5b**)

Mobile phase dichlromethane/methanol (99:1). Yield: 63%; reddish powder; ^1^H-NMR (600 MHz, DMSO/*d*_6_) δ 10.79 (s, 1H), 10.00 (s, 1H), 8.09–8.04 (m, 2H), 7.99 (dd, *J* = 7.8, 0.5 Hz, 1H), 7.87–7.84 (m, 2H), 7.71 (s, 1H), 7.34 (td, *J* = 7.7, 1.2 Hz, 1H), 6.95 (td, *J* = 7.7, 1.1 Hz, 1H), 6.90–6.87 (m, 1H), 3.25 (s, 2H), 2.52–2.50 (m, 4H), 1.48–1.43 (m, 4H), 0.87 (t, *J* = 7.4 Hz, 6H). ^13^C-NMR (150 MHz, DMSO/*d*_6_) δ 189.72, 170.60, 168.19, 144.75, 143.54, 135.89, 132.66, 131.93, 130.09, 126.61, 126.34, 121.67, 120.01, 118.76, 110.29, 58.38, 56.39, 19.85, 11.71. MS (FAB) *m*/*z* 406.4 (M+H)^+^; HRMS (FAB) calcd. for C_24_H_28_N_3_O_3_: 406.2131, found: 406.2132.

#### 6.1.6. (E)-N-(4-(2-(2-Oxoindolin-3-ylidene)acetyl)phenyl)-2-(piperidin-1-yl)acetamide (**5c**)

Mobile phase dichlromethane/methanol (98:2). Yield: 57%; reddish powder; ^1^H-NMR (600 MHz, DMSO/*d*_6_) δ 10.79 (s, 1H), 10.12 (s, 1H), 8.07 (d, *J* = 8.7 Hz, 2H), 7.99 (d, *J* = 7.7 Hz, 1H), 7.88 (d, *J* = 8.7 Hz, 2H), 7.71 (s, 1H), 7.34 (t, *J* = 7.6 Hz, 1H), 6.95 (t, *J* = 7.6 Hz, 1H), 6.89 (d, *J* = 7.8 Hz, 1H), 3.13 (s, 2H), 2.47 (s, 4H), 1.56 (dd, *J* = 10.7, 5.3 Hz, 4H), 1.40 (s, 2H). ^13^C-NMR (150 MHz, DMSO/*d*_6_) δ 189.74, 169.41, 168.20, 144.75, 143.81, 135.88, 132.64, 131.88, 129.99, 126.62, 126.36, 121.67, 120.01, 118.98, 110.30, 62.65, 54.00, 25.40, 23.50. MS (FAB) *m*/*z* 390.3 (M+H)^+^; HRMS (FAB) calcd. for C_23_H_23_N_3_O_3_: 390.1818, found: 390.1825.

#### 6.1.7. (E)-2-Morpholino-N-(4-(2-(2-oxoindolin-3-ylidene)acetyl)phenyl)acetamide (**5d**)

Mobile phase dichlromethane/methanol (98:2). Yield: 64%; reddish powder; ^1^H-NMR (600 MHz, DMSO/*d*_6_) δ 10.77 (s, 1H), 10.18 (s, 1H), 8.08–8.03 (m, 2H), 7.97 (d, *J* = 7.7 Hz, 1H), 7.88–7.84 (m, 2H), 7.70 (s, 1H), 7.33 (td, *J* = 7.7, 1.2 Hz, 1H), 6.94 (td, *J* = 7.7, 1.1 Hz, 1H), 6.90–6.87 (m, 1H), 3.65–3.62 (m, 4H), 3.19 (s, 2H), 2.53–2.51 (m, 4H). ^13^C-NMR (150 MHz, DMSO/*d*_6_) δ 189.78, 168.92, 168.21, 144.72, 143.81, 135.86, 132.66, 131.91, 129.98, 126.59, 126.38, 121.69, 120.00, 119.06, 110.31, 66.05, 62.02, 53.09. MS (FAB) *m*/*z* 392.1 (M+H)^+^; HRMS (FAB) calcd. for C_22_H_22_N_3_O_4_: 392.1610, found: 392.1589.

#### 6.1.8. (E)-2-(4-Ethylpiperazin-1-yl)-N-(4-(2-(2-oxoindolin-3-ylidene)acetyl)phenyl) acetamide (**5e**)

Mobile phase dichlromethane/methanol (95:5). Yield: 62%; reddish powder; ^1^H-NMR (600 MHz, DMSO/*d*_6_) δ 10.81 (s, 1H), 10.16 (s, 1H), 8.10–8.05 (m, 2H), 8.04–8.00 (m, 1H), 7.89–7.86 (m, 2H), 7.72 (s, 1H), 7.34 (td, *J* = 7.7, 1.2 Hz, 1H), 6.96 (td, *J* = 7.7, 1.1 Hz, 1H), 6.92–6.89 (m, 1H), 3.58 (s, 4H), 3.20 (s, 2H), 2.57 (s, 4H), 2.40 (d, *J* = 7.2 Hz, 2H), 1.01 (t, *J* = 7.2 Hz, 3H). ^13^C-NMR (150 MHz, DMSO/*d*_6_) δ 189.68, 169.03, 168.22, 144.74, 143.78, 135.93, 132.65, 131.92, 129.98, 126.65, 126.26, 121.67, 120.01, 118.98, 110.29, 61.66, 52.41, 51.99, 51.47, 11.67. MS (FAB) *m*/*z* 419.2 (M+H)^+^; HRMS (FAB) calcd. for C_24_H_27_N_4_O_3_: 419.2083, found: 419.2076.

#### 6.1.9. (E)-2-(4-Acetylpiperazin-1-yl)-N-(4-(2-(2-oxoindolin-3-ylidene)acetyl)phenyl)acetamide (**5f**)

Mobile phase dichlromethane/methanol (97:3). Yield: 60%; white solid; ^1^H-NMR (600 MHz, DMSO/*d*_6_) δ 10.79 (s, 1H), 10.23 (s, 1H), 8.07 (d, *J* = 8.8 Hz, 2H), 7.99 (d, *J* = 7.7 Hz, 1H), 7.88 (d, *J* = 8.8 Hz, 2H), 7.71 (s, 1H), 7.34 (td, *J* = 7.7, 1.0 Hz, 1H), 6.95 (td, *J* = 7.7, 0.7 Hz, 1H), 6.89 (d, *J* = 7.8 Hz, 1H), 3.50 (d, *J* = 4.3 Hz, 4H), 3.26 (s, 2H), 2.56 (s, 2H), 2.50 (s, 2H), 2.00 (s, 3H). ^13^C-NMR (150 MHz, DMSO/*d*_6_) δ 189.75, 168.20, 168.12, 144.75, 143.81, 135.89, 132.66, 131.93, 129.99, 126.62, 126.34, 121.68, 120.01, 119.06, 110.30, 61.36, 52.74, 52.31, 45.50, 40.67, 21.13. MS (FAB) *m*/*z* 433.1 (M+ H)^+^; HRMS (FAB) calcd. for C_24_H_25_N_4_O_4_: 433.1876, found: 433.1890.

#### 6.1.10. Tert-butyl (E)-4-(2-oxo-2-((4-(2-(2-oxoindolin-3-ylidene)acetyl)phenyl)amino) ethyl) piperazine-1-carboxylate (**5g**)

Mobile phase dichlromethane/methanol (99:1). Yield: 67%; reddish powder; ^1^H-NMR (600 MHz, CDCl_3_) δ 9.33 (s, 1H), 8.29 (d, *J* = 7.6 Hz, 1H), 8.23 (s, 1H), 8.14–8.10 (m, 2H), 7.84 (s, 1H), 7.75–7.72 (m, 2H), 7.31 (td, *J* = 7.7, 1.2 Hz, 1H), 7.01 (td, *J* = 7.7, 0.9 Hz, 1H), 6.86 (d, *J* = 7.7 Hz, 1H), 3.54 (s, 4H), 3.20 (s, 2H), 2.60 (s, 4H), 1.48 (s, 9H). ^13^C-NMR (150 MHz, CDCl_3_) δ 189.58, 169.30, 168.42, 154.63, 143.25, 142.40, 136.49, 133.43, 132.64, 130.46, 128.06, 126.47, 122.87, 120.76, 118.98, 110.08, 80.21, 62.14, 53.34, 28.41. MS (FAB) *m*/*z* 491.2 (M+H)^+^; HRMS (FAB) calcd. for C_27_H_31_N_4_O_5_: 491.2294, found: 491.2266.

#### 6.1.11. (E)-N-(4-(2-(2-Oxoindolin-3-ylidene)acetyl)phenyl)-2-(4-(pyrimidin-2-yl)piperazin-1-yl)acetamide (**5h**)

Mobile phase dichlromethane/methanol (99:1). Yield: 61%; reddish powder;^1^H-NMR (600 MHz, DMSO/*d*_6_) δ 10.78 (s, 1H), 10.23 (s, 1H), 8.36 (d, *J* = 4.7 Hz, 2H), 8.10–8.03 (m, 2H), 7.98 (dd, *J* = 7.7, 0.6 Hz, 1H), 7.91–7.85 (m, 2H), 7.70 (s, 1H), 7.33 (td, *J* = 7.7, 1.2 Hz, 1H), 6.95 (td, *J* = 7.6, 1.0 Hz, 1H), 6.90–6.86 (m, 1H), 6.63 (t, *J* = 4.7 Hz, 1H), 3.84–3.78 (m, 4H), 3.25 (s, 2H), 2.62–2.57 (m, 4H). ^13^C-NMR (150 MHz, DMSO/*d*_6_) δ 189.78, 169.04, 168.20, 161.21, 157.89, 144.74, 143.85, 135.87, 132.65, 131.92, 130.00, 126.60, 126.39, 121.68, 120.01, 119.07, 110.30, 110.09, 61.70, 52.41, 43.21. MS (FAB) *m*/*z* 469.2 (M+H)^+^; HRMS (FAB) calcd. for C26H25N6O3: 469.2006, found: 469.2639.

### 6.2. Biology

#### 6.2.1. Evaluation of In Vitro Antiproliferative Activity for Compounds by NCI

The methodology of the NCI anticancer screening has been described in detail elsewhere (http://www.dtp.nci.nih.gov accessed on 18 November 2022). Primary anticancer assay was performed at approximately 60 human tumor cell lines panel derived from nine neoplastic diseases, in accordance with the protocol of the Drug Evaluation Branch, National Cancer Institute, Bethesda, USA [41].

#### 6.2.2. Evaluation of Antiproliferative Activity for Compounds against HL-60 and PBMC

The synthesized compounds and staurosporine (AdipoGen Life Sciences, San Diego, CA, USA) were dissolved in dimethyl sulfoxide (DMSO/*d*_6_) (FUJIFILM-Wako, Osaka, Japan), and the solution was added to the cell-culture medium at a 1:100 volume. The human promyelocytic leukemia cells line HL-60 (provided by the RIKEN BRC through the National Bio-Resource Project of the MEXT/AMED, Japan (RCB0041 and RCB1904)) was maintained in RPMI-1640, supplemented with 10% heat-inactivated fetal bovine serum (FBS) (Capricorn Scientific, Ebsdorfergrund, Germany), 89 μg/mL of streptomycin (Meiji Seika Pharma, Tokyo, Japan) and 2.0 μg/mL of amphotericin B (Clinigen, Burton-on-Trent, UK). PBMCs from a healthy donor were obtained from Precision for Medicine (Frederick, MD, USA), and maintained in RPMI-1640 supplemented with 10%FBS, 89 μg/mL of streptomycin and 2.0 μg/mL of amphotericin B. To suspended HL-60 and PBMC cells, chemicals were immediately added. At 2 d post-addition, MTT assay was performed to evaluate cell viability as previously described [52]. IC_50_ was calculated using GraphPad Prism 9.4.1.

#### 6.2.3. Cell Cycle Analysis and Apoptosis Analysis

HL-60 cells were plated at 8 × 10^4^ cells on a 35 mm dish (Sumitomo Bakelite, Tokyo, Japan) and chemicals were added. To observe the cell cycle, the cells were incubated for 1 d, and washed with PBS (500 µL) followed by incubation with Cell Cycle Assay Solution Deep Red (Dojindo, Kumamoto, Japan) at 37 °C for 15 min. Fluorescence signal was detected in FL4 using flow cytometer BD FACSCalibur (BD Biosciences, Franklin Lakes, NJ, USA). For apoptosis/necrosis assay, the cells were incubated for 0.5–1 d, and washed with PBS followed by incubation with annexin binding buffer containing Annexin-V-FITC (MBL Life Science, Tokyo, Japan) and 7-Aminoactinomycin D (7-AAD) (BD Biosciences) at room temperature for 15 min. Fluorescence signals of FITC and 7-AAD were detected in FL1 and FL3, respectively, using flow cytometer BD FACSCalibur.

#### 6.2.4. Western Blot

Western blot analysis was conducted basically as described previously [53]. Differences were as follows: total protein per well for gel electrophoresis was 3 μg. As an antibody, Phospho-p44/42 MAPK (Erk1/2) (Thr202/Tyr204) (D13.14.4E) XP^®^ Rabbit mAb (Cell Signaling Technology) or anti-β-actin (AC-15) (Sigma–Aldrich) were used.

### 6.3. In Silico Screening

#### 6.3.1. Molecular Docking

The 3D X-ray crystal structure of ERK2 co-crystalized with SCH772984 was downloaded from the protein data bank, PDB, (PDB ID: 6GDM). Docking of the target ligands as a conformations database was performed inside the SCH772984 binding site using MOE 2019 as described before [54,55]. A scoring function, London dG, was employed for comparison of different conformers; poses with lower values are more beneficial. Re-docking of the native ligand SCH772984 on the active site of ERK (PDB ID: 6GDM) was performed for docking protocol validation.

#### 6.3.2. Prediction of Physicochemical Properties and Pharmacokinetics

The physicochemical and pharmacokinetic properties of compounds (**5a**–**h**) and sunitinib were estimated by the SwissADME web tool [50,51,56].

## Figures and Tables

**Table 1:** In vitro growth inhibition and lethality obtained from the single dose (10 µM) test of compounds **5a**–**h**.

Panel	Cell Line	Growth Inhibition (GI) Percent at 10 µM							
		5a	5b	5c	5d	5e	5f	5g	5h
Leukemia	CCRF-CEM	−0.58	−2.47	−25.57	−11.86	95.63	−4.06	−22.12	−22.54
	HL-60 (TB)	−45.21	−43.91	−46.85	−32.82	−42.41	92.45	−46.44	87.43
	K-562	−25.82	−35.03	99.15	97.72	−14.55	75.31	−22.75	73.96
	MOLT-4	−22.48	−31.26	−20.73	−16.45	97.87	96.34	−44.85	96.53
	RPMI-8226	−13.65	−26.17	99.56	87.8	89.17	51.88	−28.80	57.83
Non-Small Cell Lung Cancer	A549/ATCC	7.65	15.65	5.75	8.73	5.40	3.91	25.07	-
	EKVX	64.47	97.33	58.57	52.43	55.57	34.27	−20.50	29.54
	HOP-62	2.47	5.92	10.48	4.68	1.90	1.32	13.62	2.03
	HOP-92	72.22	−30.46	51.94	34.82	61.49	16.95	−40.81	6.74
	NCI-H226	90.40	−32.95	68.41	62.8	89.46	20.85	−75.77	30.22
	NCI-H23	31.40	49.02	35.91	25.51	28.69	14.27	87.95	13.07
	NCI-H322M	7.28	2.41	9.22	8.40	1.08	2.44	13.76	-
	NCI-H460	22.85	32.52	27.57	13.56	17.94	-	31.57	5.68
	NCI-H522	−51.49	−75.35	65.98	72.97	89.06	64.81	−81.87	50.89
Colon Cancer	COLO 205	−44.48	−68.73	76.69	72.46	96.60	25.69	−94.90	25.62
	HCC-2998	-	-	-	-	-	-	14.35	-
	HCT-116	−88.52	−88.15	−75.60	−23.15	−66.61	55.51	−96.98	58.67
	HCT-15	−10.90	−51.56	−17.35	91.63	90.36	31.60	−61.56	28.88
	HT29	−32.52	−47.12	79.2	51.52	80.76	19.20	−50.13	13.02
	KM12	4.34	17.72	5.98	4.3	7.44	-	26.35	-
	SW-620	−62.38	−76.08	−11.10	97.61	−17.31	52.79	−88.72	58.93
CNS Cancer	SF-268	27.17	25.01	6.29	21.48	24.54	-	34.98	7.61
	SF-295	-	3.44	4.19	1.03	-	-	13.25	-
	SF-539	58.59	−35.29	51.98	33.45	47.06	19.08	−72.33	18.57
	SNB-19	18.59	29.18	16.02	13.30	14.21	5.03	29.92	1.38
	SNB-75	24.89	32.00	8.81	23.84	24.28	-	-	38.98
	U251	20.79	36.19	16.26	13.18	13.21	1.68	32.48	4.09
Melanoma	LOX IMVI	−61.45	−98.96	−59.39	−32.88	−33.15	55.70	−100	59.67
	MALME-3M	−51.93	−89.79	−11.41	−30.57	−30.35	98.15	−81.40	80.97
	M14	55.53	−57.59	43.48	36.56	41.68	21.33	−48.57	23.73
	MDA-MB-435	18.23	32.15	16.21	14.82	16.54	0.65	28.10	5.04
	SK-MEL-2	1.35	26.27	11.26	3.60	-	3.62	29.01	5.84
	SK-MEL-28	26.14	76.26	23.69	10.76	9.36	4.05	−7.77	5.89
	SK-MEL-5	22.84	29.75	16.39	16.76	12.88	0.23	92.07	2.24
	UACC-257	35.99	40.23	26.66	18.85	23.92	13.54	74.24	14.06
	UACC-62	44.45	64.56	40.53	40.72	28.94	27.21	78.70	22.70
Ovarian cancer	IGROV1	20.65	40.83	16.87	14.78	11.68	-	74.06	-
	OVCAR-3	−45.16	−72.98	−32.20	−35.23	−31.09	56.64	−83.94	51.82
	OVCAR-4	45.75	48.46	30.51	25.41	37.02	10.47	−7.98	13.22
	OVCAR-5	30.72	38.39	28.57	23.16	27.63	6.67	−33.88	-
	OVCAR-8	42.34	−48.41	41.34	26.49	27.92	1.19	−39.58	9.37
	NCI/ADR-RES	13.50	74.59	40.33	10.56	11.44	0.23	92.02	2.54
	SK-OV-3	1.01	6.23	11.06	8.00	1.87	-	6.75	4.52
Renal Cancer	786–0	97.75	−87.01	42.78	30.65	61.43	17.68	−90.34	17.63
	A498	-	-	-	-	-	-	-	-
	ACHN	−27.51	−97.53	98.59	97.21	−23.79	24.89	−97.14	21.01
	CAKI-1	66.71	87.63	39.91	42.13	59.4	28.32	−20.53	21.56
	RXF 393	−50.71	−93.84	99.46	81.83	−10.63	45.27	−96.23	42.73
	SN12C	47.76	71.39	42.9	30.75	51.07	22.72	−37.69	11.87
	TK-10	41.12	−27.68	5.04	5.76	29.77	-	−25.96	-
Prostate Cancer	PC-3	20.15	68.93	48.74	9.24	16.48	4.42	74.73	14.82
	DU-145	68.03	93.75	32.78	48.07	43.57	-	92.09	3.35
Breast Cancer	MCF7	97.26	−26.39	88.56	77.09	86.96	74.61	−86.32	66.02
	HS 578T	28.86	39.6	30.69	22.04	24.84	12.17	61.59	14.34
	BT-549	17.90	40.49	22.76	-	10.65	-	−21.27	4.81
	T-47D	59.34	−12.28	53.94	46.40	60.89	43.43	−66.39	47.61
	MDA-MB-468	68.69	76.47	57.59	51.50	53.78	49.41	−67.01	50.04
Mean		63.66	90.35	52.52	44.76	51.47	23.22	−8.44	24.36

**Table 2:** In vitro cytotoxicity IC_50_ (µM) results against acute myeloid leukemia cells HL-60 PBMC and selectivity index (SI) which was calculated as the ratio of cytotoxicity (IC_50_) of PBMC to cancer cells (HL-60).

Cells	5a	5b	5c	5d	5e	5f	5g	5h	Staurosporine
HL60	0.72 ± 0.09	0.38 ± 0.08	1.06 ± 0.10	1.43 ± 0.16	1.07 ± 0.13	2.57 ± 0.29	0.57 ± 0.05	0.79 ± 0.08	0.013 ± 0.002
PBMC	-	14.17 ± 1.13	-	-	-	-	-	-	-
SI	-	37.2	-	-	-	-	-	-	-

All IC_50_ values are the mean ± standard error of three different experiments. -: not tested.

**Table 3:** Effect of compounds **5b** and **5g** on cell cycle distribution in HL-60 cells.

Sample	Cell Cycle Distribution (%)		
	%G0-G1	%S	%G2/M
DMSO/HL-60	61	16.7	21.5
**5b**/HL-60	68.8	10.3	20.1
**5g**/HL-60	67.5	15.6	16.7

**Table 4:** Physicochemical properties of the synthesized compounds **5a**–**h** compared to sunitinib.

	5a	5b	5c	5d	5e	5f	5g	5h	Sunitinib
Mwt	377.44	405.49	389.45	391.42	418.49	432.47	490.55	468.51	398.47
Fraction Csp3	0.23	0.29	0.26	0.23	0.29	0.25	0.33	0.19	0.36
Num. rotatable bonds	8	10	6	6	7	7	9	7	8
Num. H-bond acceptors	4	4	4	5	5	5	6	6	4
Num. H-bond donors	2	2	2	2	2	2	2	2	3
Molar Refractivity	113.01	122.63	119.62	115.90	131.24	131.44	147.37	142.26	116.31
TPSA	78.51	78.51	78.51	87.74	81.75	98.82	108.05	107.53	77.23
Log P	2.25	2.61	3.05	2.75	3.24	2.82	3.57	2.83	3.55
GI absorption	High	High	High	High	High	High	High	High	High
BBB penetration	No	Yes	No	No	No	No	No	No	Yes
Lipinski violation	0	0	0	0	0	0	0	0	0
Bioavailability score	0.55	0.55	0.55	0.55	0.55	0.55	0.55	0.55	0.55

## Data Availability

Data is contained within the article and Supplementary Materials.

## Supplementary Materials

The following supporting information can be downloaded at: https://www.mdpi.com/article/10.3390/ph15121579/s1, Figure S1: ^1^H-NMR spectrum of compound **3** (600 MHz, DMSO/*d*_6_); Figure S2: ^13^C-NMR spectrum of compound **3** (150 MHz, DMSO/*d*_6_); Figure S3: ^1^H-NMR spectrum of compound **4** (600 MHz, DMSO-/*d*_6_); Figure S4: ^13^C-NMR spectrum of compound **4** (150 MHz, DMSO/*d*_6_); Figure S5: ^1^H-NMR spectrum of compound **5a** (600 MHz, DMSO/*d*_6_); Figure S6: ^13^C-NMR spectrum of compound **5a** (150 MHz, DMSO/*d*_6_); Figure S7: ^1^H-NMR spectrum of compound **5b** (600 MHz, DMSO/*d*_6_); Figure S8: ^13^C-NMR spectrum of compound **5b** (150 MHz, DMSO/*d*_6_); Figure S9: ^1^H-NMR spectrum of compound **5c** (600 MHz, DMSO/*d*_6_); Figure S10: ^13^C-NMR spectrum of compound **5c** (150 MHz, DMSO/*d*_6_); Figure S11: ^1^H-NMR spectrum of compound **5d** (600 MHz, CDCl_3_); Figure S12: ^13^C-NMR spectrum of compound **5d** (150 MHz, CDCl_3_); Figure S13: ^1^H-NMR spectrum of compound **5e** (600 MHz, DMSO/*d*_6_); Figure S14: ^13^C-NMR spectrum of compound **5e** (150 MHz, DMSO/*d*_6_); Figure S15: ^1^H-NMR spectrum of compound **5f** (600 MHz, DMSO/*d*_6_); Figure S16: ^13^C-NMR spectrum of compound **5f** (150 MHz, DMSO/*d*_6_); Figure S17: ^1^H-NMR spectrum of compound **5g** (600 MHz, DMSO/*d*_6_); Figure S18: ^13^C-NMR spectrum of compound **5g** (150 MHz, DMSO/*d*_6_); Figure S19: ^1^H-NMR spectrum of compound **5h** (600 MHz, DMSO/*d*_6_); Figure S20: ^13^C-NMR spectrum of compound **5h** (150 MHz, DMSO/*d*_6_); Figure S21: One-dose mean graph of compound **5a** against different cancer cell lines based on Sulforhodamine B (SRB) assay at NCI at 10 µM concentration after 48 h; Figure S22: One-dose mean graph of compound 5b against different cancer cell lines based on SRB assay at NCI at 10 µM concentration after 48 h; Figure S23: One-dose mean graph of compound 5c against different cancer cell lines based on SRB assay at NCI at 10 µM concentration after 48 h; Figure S24: One-dose mean graph of compound **5d** against different cancer cell lines based on Sulforhodamine B (SRB) assay at NCI at 10 µM concentration after 48 h; Figure S25: One-dose mean graph of compound **5e** against different cancer cell lines based on SRB assay at NCI at 10 µM concentration after 48 h; Figure S26: One-dose mean graph of compound **5f** against different cancer cell lines based on SRB assay at NCI at 10 µM concentration after 48 h; Figure S27: One-dose mean graph of compound **5g** against different cancer cell lines based on Sulforhodamine B (SRB) assay at NCI at 10 µM concentration after 48 h; Figure S28: One-dose mean graph of compound **5h** against different cancer cell lines based on SRB assay at NCI at 10 µM concentration after 48 h.
