# Supplementary material for: New Insights into the Structural Requirements of Isatin-Derived Pro-Apoptotic Agents against Acute Myeloid Leukemia
Source: Pharmaceuticals (Basel). 2022 Dec 17;15(12):1579. doi: 10.3390/ph15121579 (PMC9784816; doi:10.3390/ph15121579)
Supplement: Supplementary file 1 [file pharmaceuticals-15-01579-s001.zip › pharmaceuticals-2093347-supplementary.pdf]

Supplementary Data:

New Insights into the Structural Requirements of Isatin-derived Pro-apoptotic Agents against Acute Myeloid Leukemia

Ahmed K. Hamdy [1,2], Takashi Sakamoto [1], Tsugumasa Toma [1], Masaharu Sakamoto [1], Mohammed A. S. Abourehab[3], Masami Otsuka [1,4], Mikako Fujita [1*], Hiroshi Tateishi [1*] and Mohamed O. Radwan [1,5*]

[1] Medicinal and Biological Chemistry Science Farm Joint Research Laboratory, Faculty of Life Sciences, Kumamoto University, Kumamoto 862-0973, Japan

[2] Department of Medicinal Chemistry, Faculty of Pharmacy, Assiut University, Assiut 71526, Egypt

[3] Department of Pharmaceutics, College of Pharmacy, Umm Al-Qura University, Makkah 21955, Saudi Arabia

[4] Department of Drug Discovery, Science Farm, Ltd., Kumamoto 862-0976, Japan

[5] National Research Centre, Chemistry of Natural Compounds Department, Pharmaceutical and Drug Industries Research Division, Dokki, Cairo 12622, Egypt

* Correspondence: mohamedradwan@kumamoto-u.ac.jp (M.O.R.); htateishi@kumamoto-u.ac.jp (H.T.); mfujita@kumamoto-u.ac.jp (M.F.)

Table of contents

Figure	Title	Page
**S1**	^{1}H-NMR spectrum of compound **3** (600MHz, DMSO/d_6)	**2**
**S2**	^{13}C-NMR spectrum of compound **3** (150MHz, DMSO/d_6)	**2**
**S3**	^{1}H-NMR spectrum of compound **4** (600MHz, DMSO/d_6)	**3**
**S4**	^{13}C-NMR spectrum of compound **4** (150MHz, DMSO/d_6)	**3**
**S5**	^{1}H-NMR spectrum of compound **5a** (600MHz, DMSO/d_6)	**4**
**S6**	^{13}C-NMR spectrum of compound **5a** (150MHz, DMSO/d_6)	**4**
**S7**	^{1}H-NMR spectrum of compound **5b** (600MHz, DMSO/d_6)	**5**
**S8**	^{13}C-NMR spectrum of compound **5b** (150MHz, DMSO/d_6)	**5**
**S9**	^{1}H-NMR spectrum of compound **5c** (600MHz, DMSO/d_6)	**6**
**S10**	^{13}C-NMR spectrum of compound **5c** (150MHz, DMSO/d_6)	**6**
**S11**	^{1}H-NMR spectrum of compound **5d** (600MHz, DMSO/d_6)	**7**
**S12**	^{13}C-NMR spectrum of compound **5d** (150MHz, DMSO/d_6)	**7**
**S13**	^{1}H-NMR spectrum of compound **5e** (600MHz, DMSO/d_6)	**8**
**S14**	^{13}C-NMR spectrum of compound **5e** (150MHz, DMSO/d_6)	**8**
**S15**	^{1}H-NMR spectrum of compound **5f** (600MHz, DMSO/d_6)	**9**
**S16**	^{13}C-NMR spectrum of compound **5f** (150MHz, DMSO/d_6)	**9**
**S17**	^{1}H-NMR spectrum of compound **5g** (600MHz, $CDCl_3$)	**10**
**S18**	^{13}C-NMR spectrum of compound **5g** (150MHz, $CDCl_3$)	**10**
**S19**	^{1}H-NMR spectrum of compound **5h** (600MHz, DMSO/d_6)	**11**
**S20**	^{13}C-NMR spectrum of compound **5h**(150MHz, DMSO/d_6)	**11**
**S21**	NCI SRB assay mean graph of compound **5a** at 10 μM concentration.	**12**
**S21**	NCI SRB assay mean graph of compound **5b** at 10 μM concentration.	**13**
**S23**	NCI SRB assay mean graph of compound **5c** at 10 μM concentration.	**14**
**S24**	NCI SRB assay mean graph of compound **5d** at 10 μM concentration.	**15**
**S25**	NCI SRB assay mean graph of compound **5e** at 10 μM concentration.	**16**
**S26**	NCI SRB assay mean graph of compound **5f** at 10 μM concentration.	**17**
**S27**	NCI SRB assay mean graph of compound **5g** at 10 μM concentration.	**18**
**S28**	NCI SRB assay mean graph of compound **5h** at 10 μM concentration.	**19**

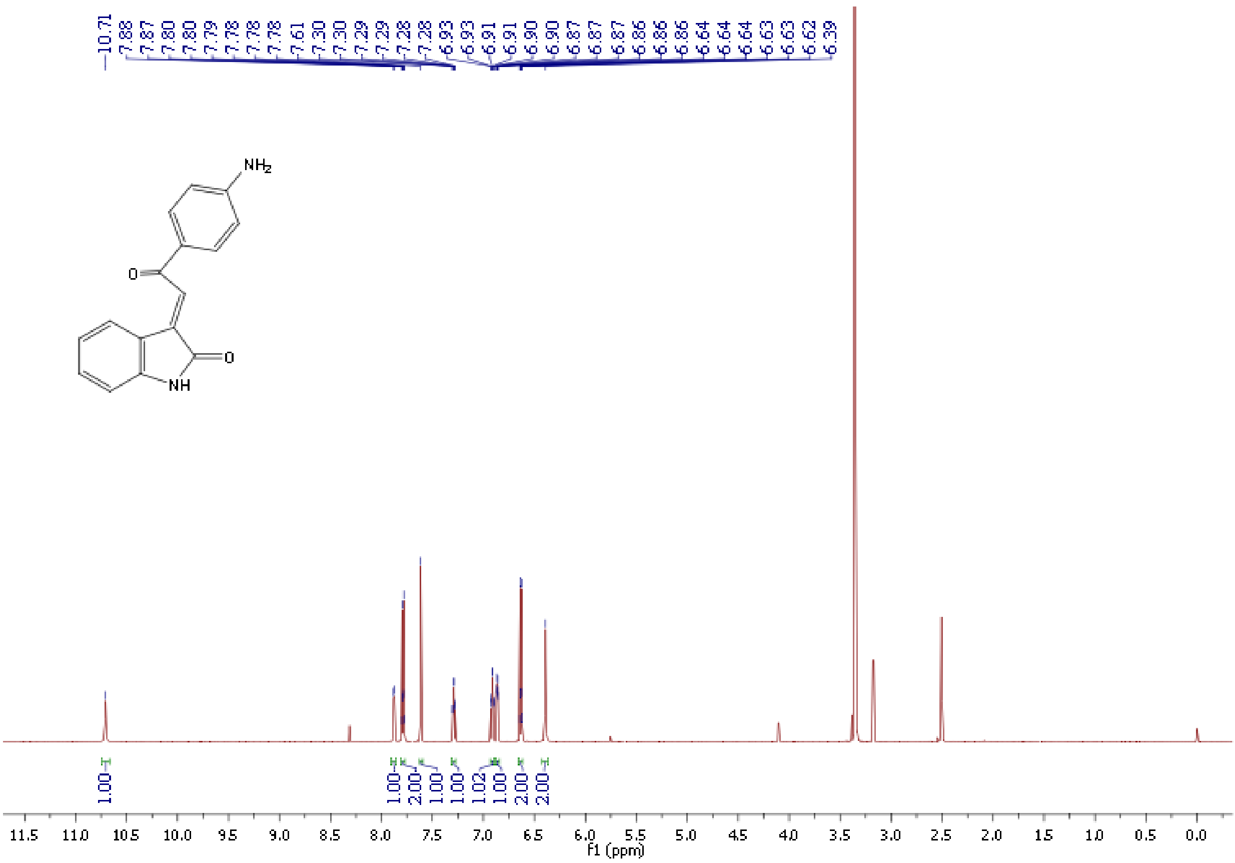

Figure S1: ^{1}H-NMR spectrum of compound **3** (600 MHz, DMSO/d_6)

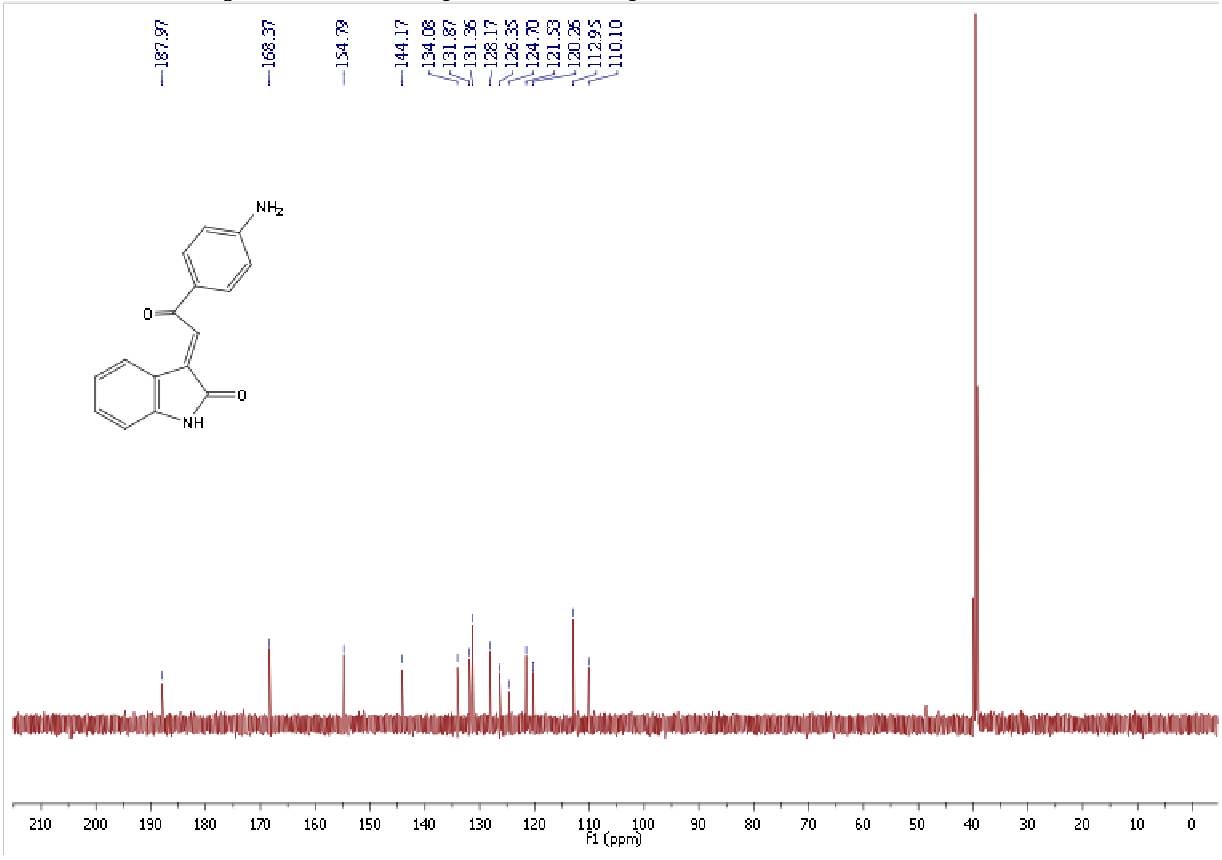

Figure S2: ^{13}C-NMR spectrum of compound **3** (150 MHz, DMSO/d_6)

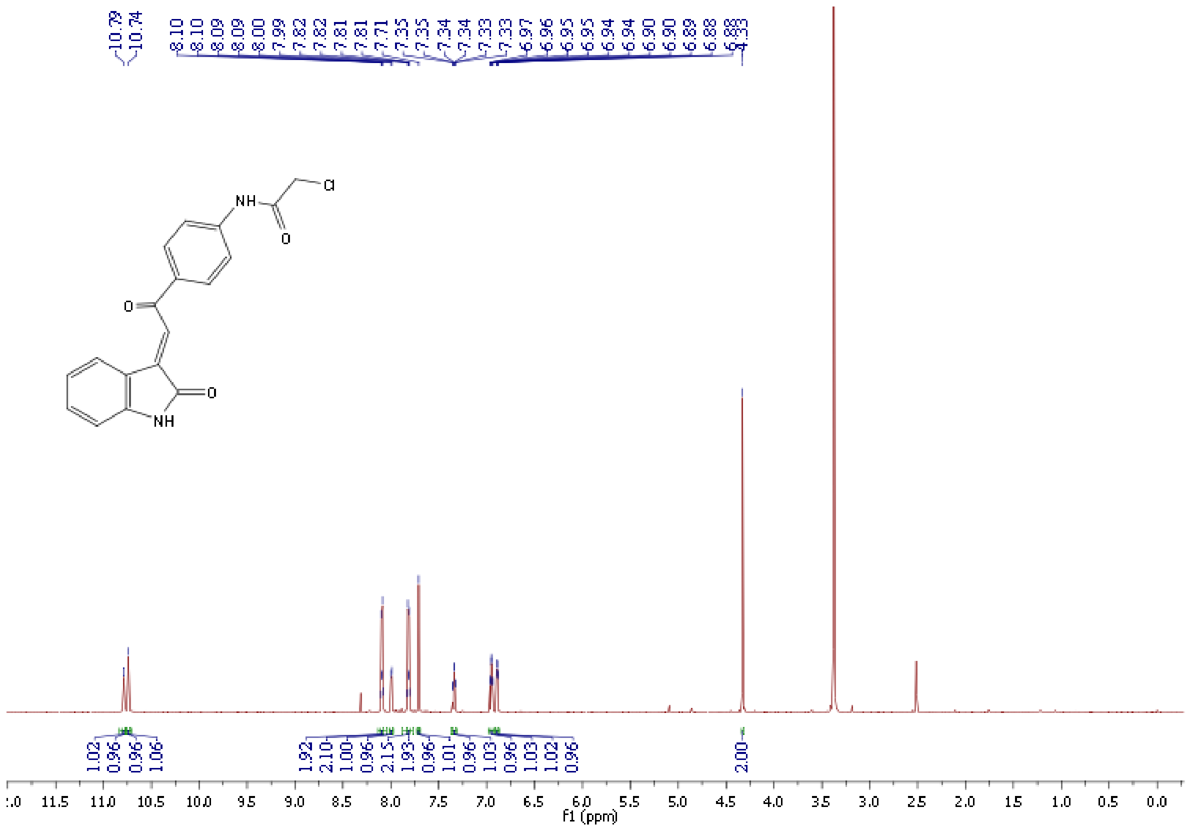

Figure S3: ^{1}H-NMR spectrum of compound **4** (600 MHz, DMSO/d_6)

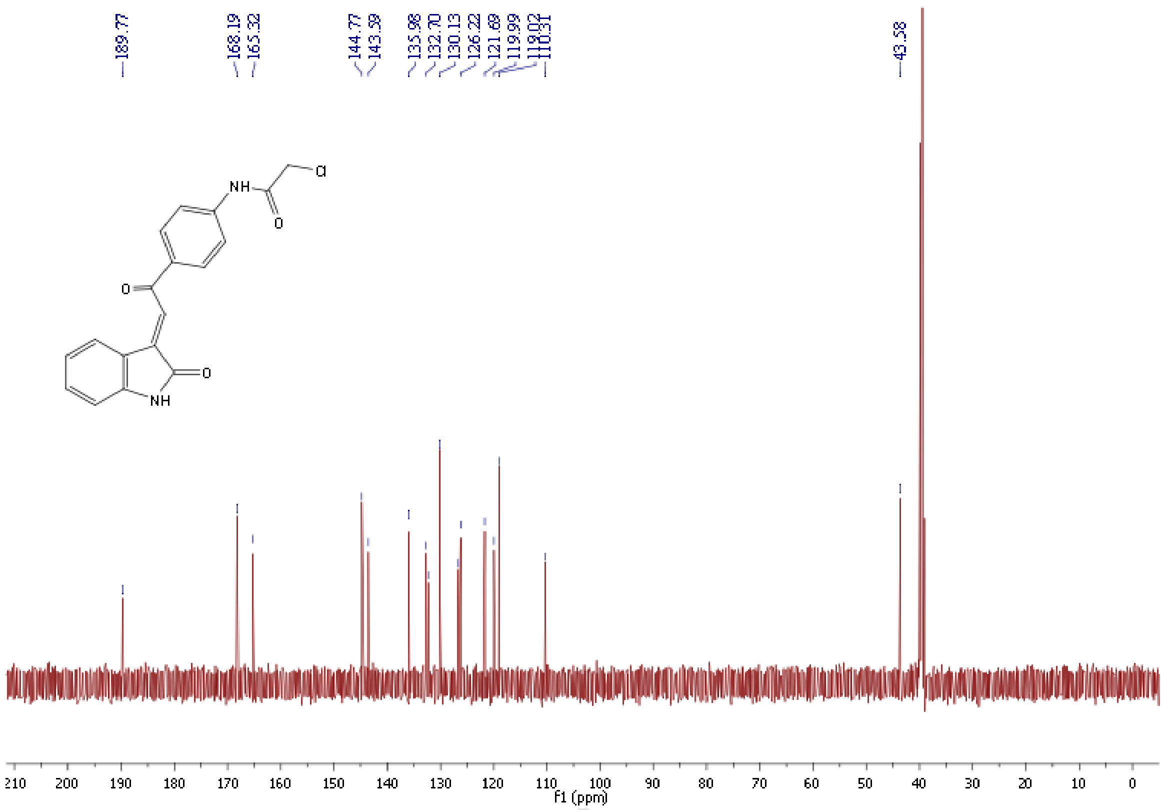

Figure S4: ^{13}C-NMR spectrum of compound **4** (150 MHz, DMSO/d_6)

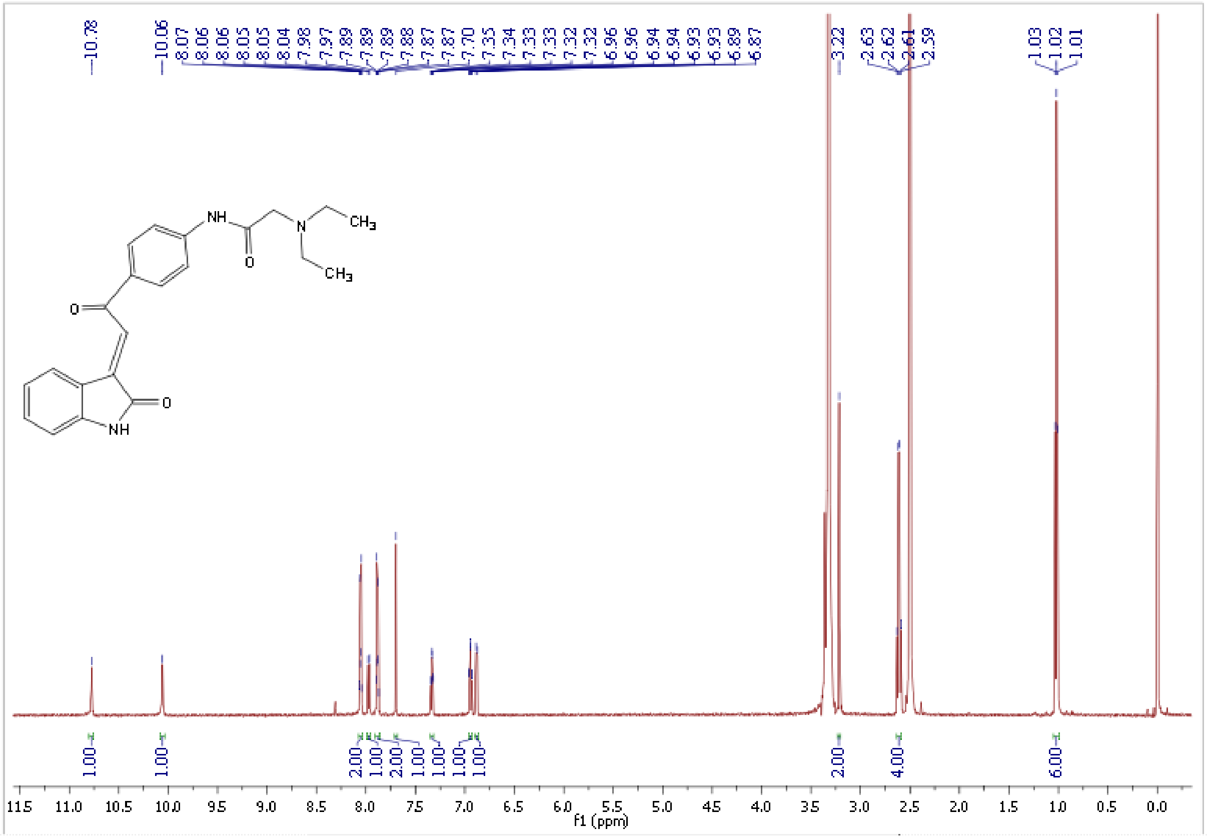

Figure S5: ^{1}H-NMR spectrum of compound **5a** (600 MHz, DMSO/d_6)

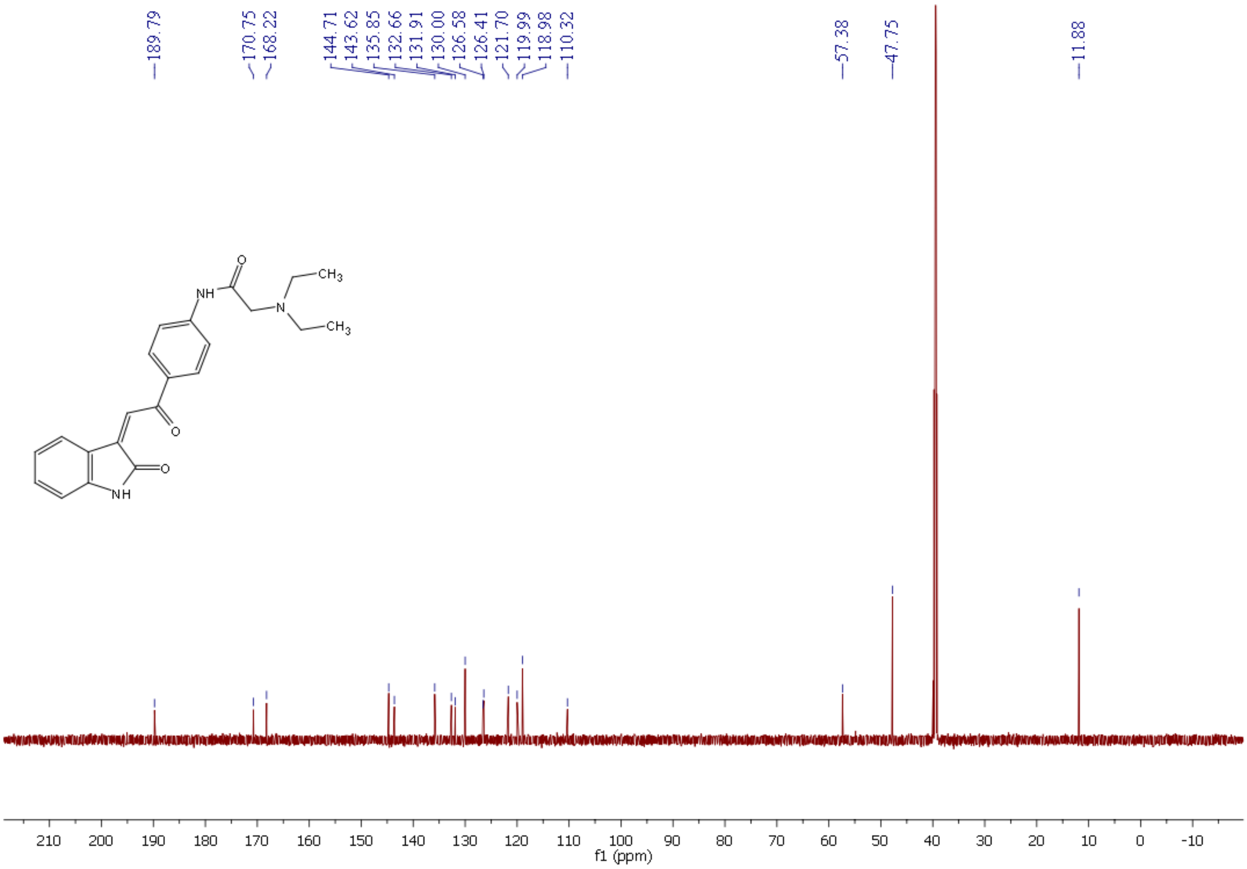

Figure S6: ^{13}C-NMR spectrum of compound **5a** (150 MHz, DMSO/d_6)

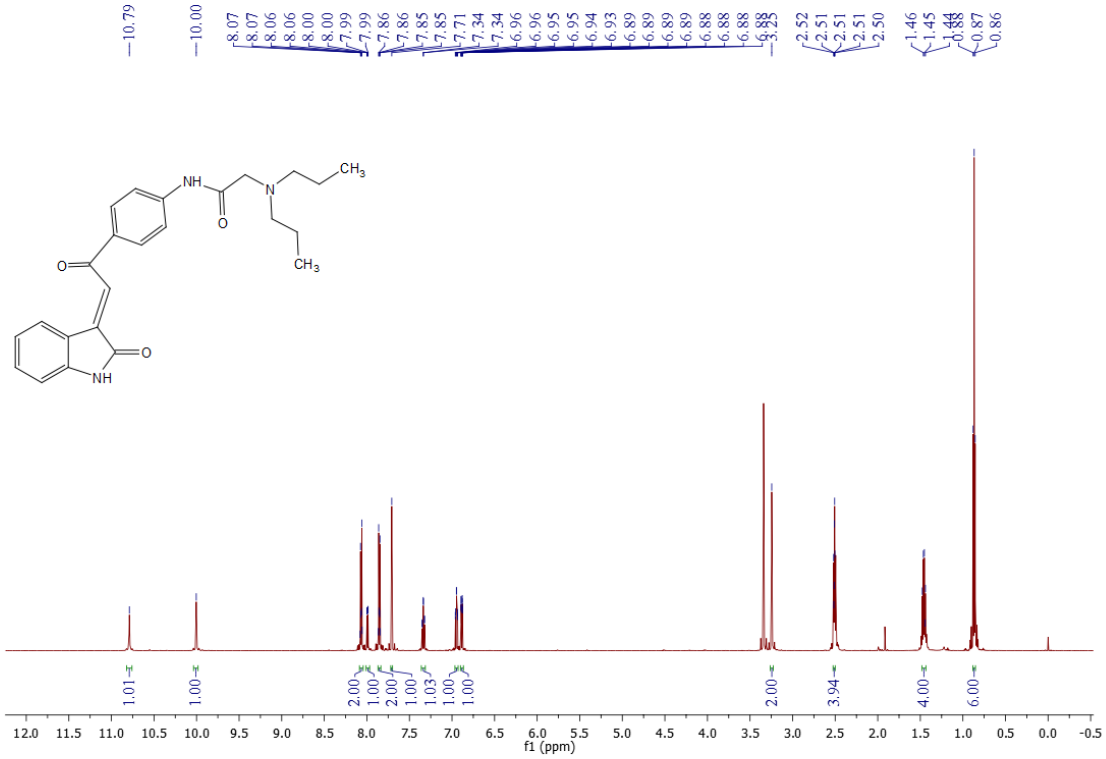

Figure S7: ^{1}H-NMR spectrum of compound **5b** (600 MHz, DMSO/*d*$_6$)

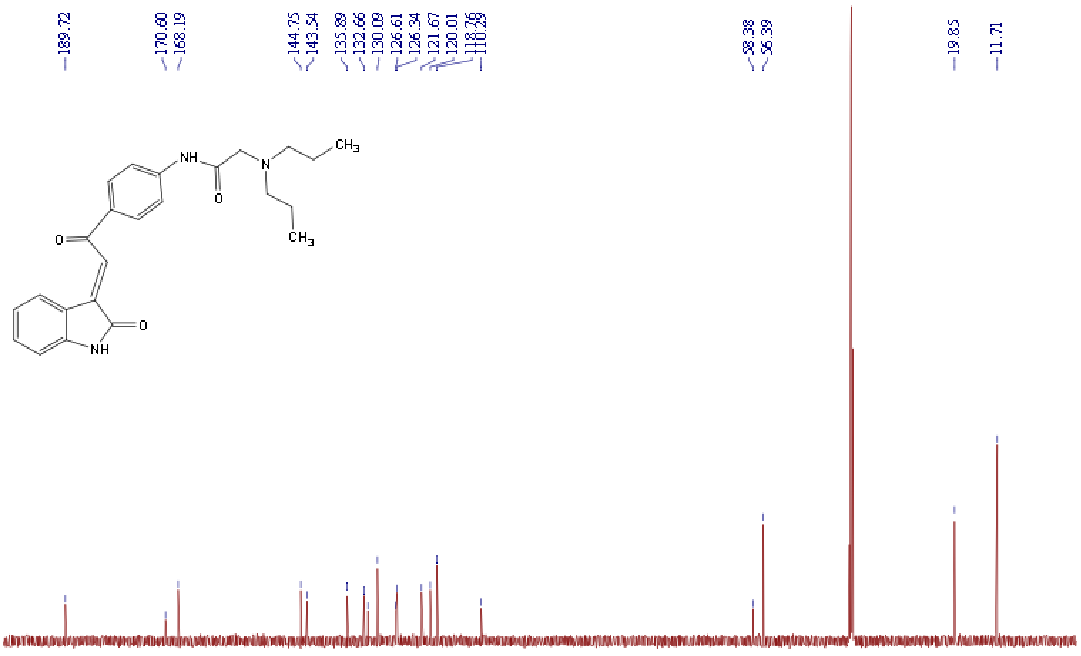

Figure S8: ^{13}C-NMR spectrum of compound **5b** (150 MHz, DMSO/*d*$_6$)

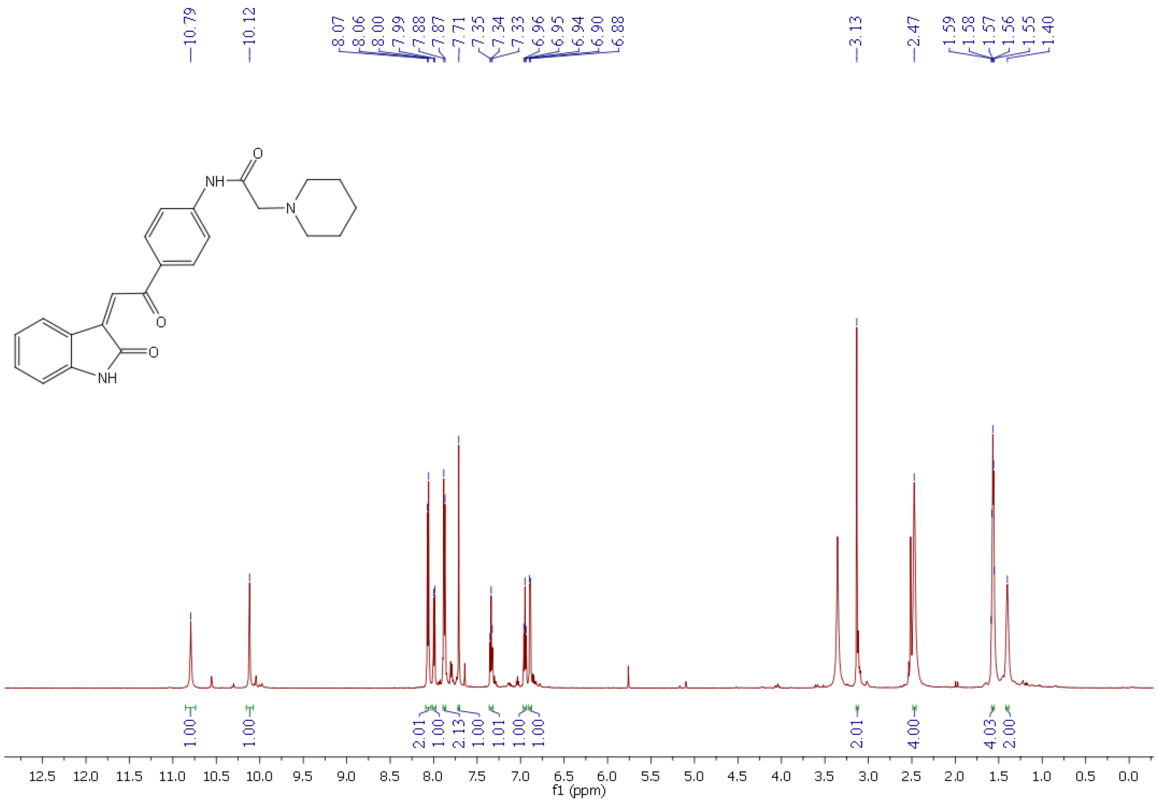

Figure S9: ^{1}H-NMR spectrum of compound **5c** (600 MHz, DMSO/d_6)

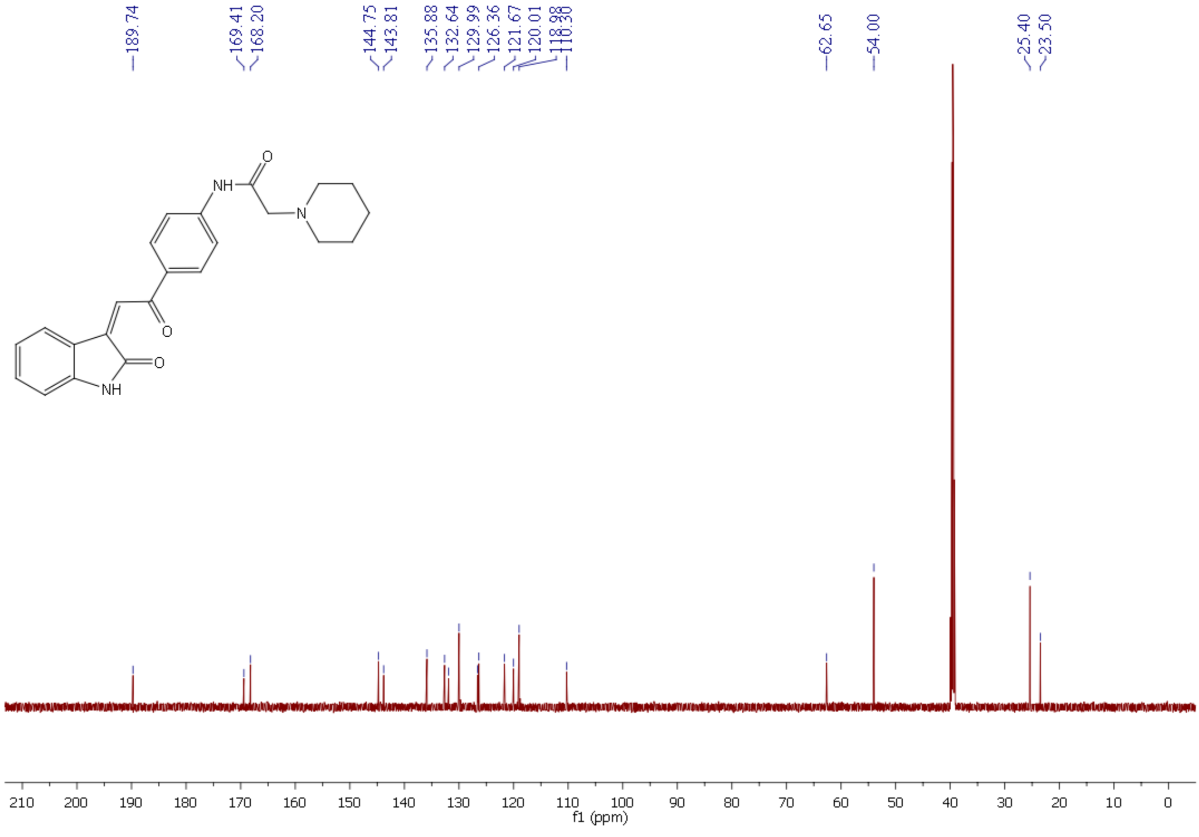

Figure S10: ^{13}C-NMR spectrum of compound **5c** (150 MHz, DMSO/d_6)

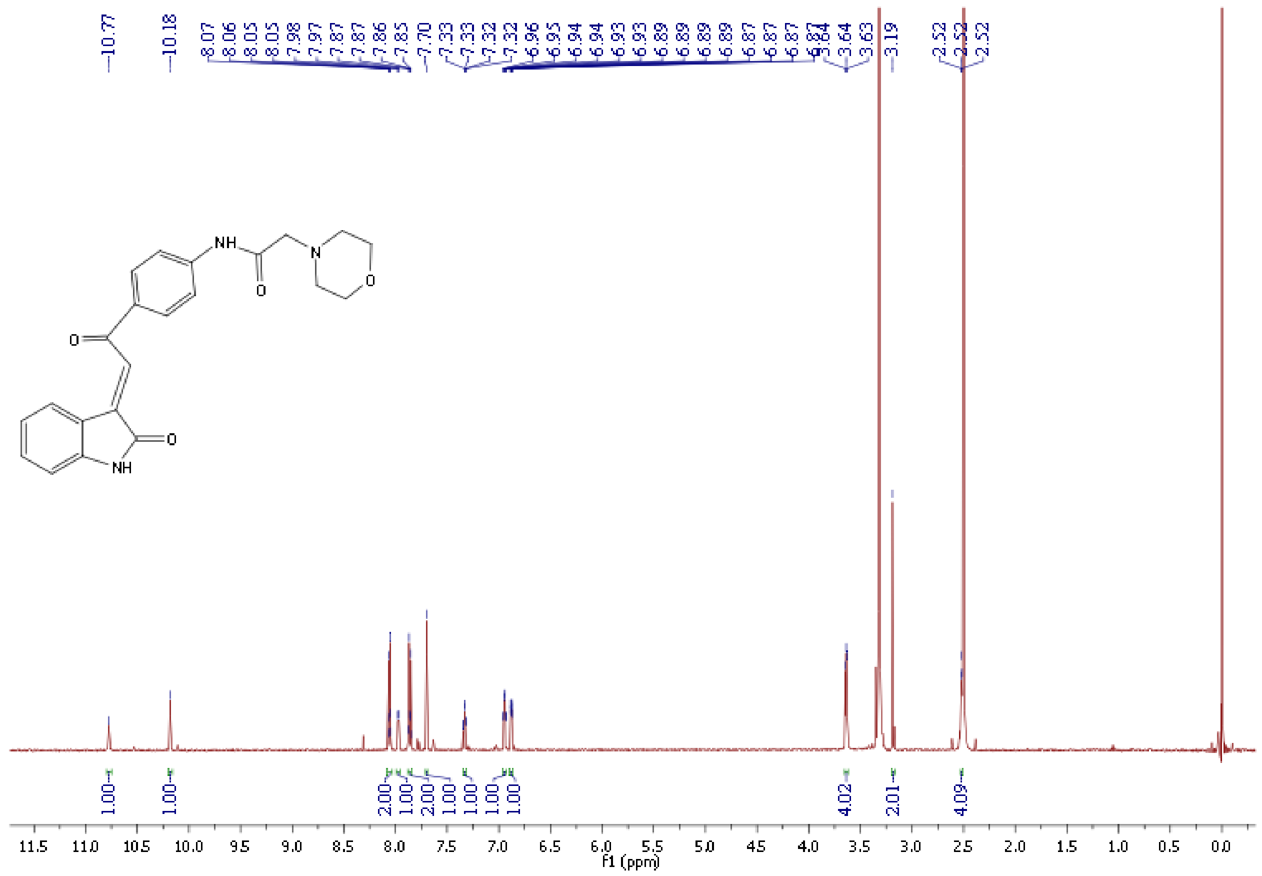

Figure S11: ^{1}H-NMR spectrum of compound **5d** (600 MHz, DMSO/d_6)

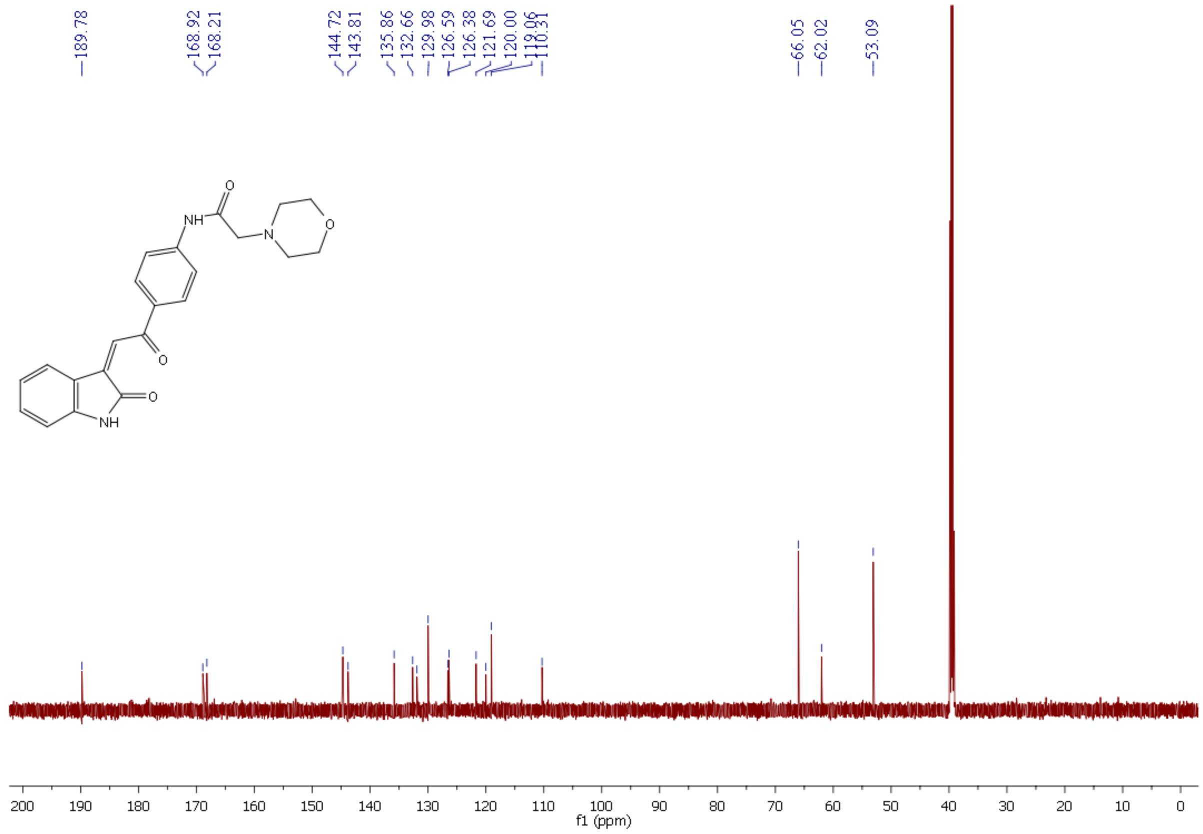

Figure S12: ^{13}C-NMR spectrum of compound **5d** (150 MHz, DMSO/d_6)

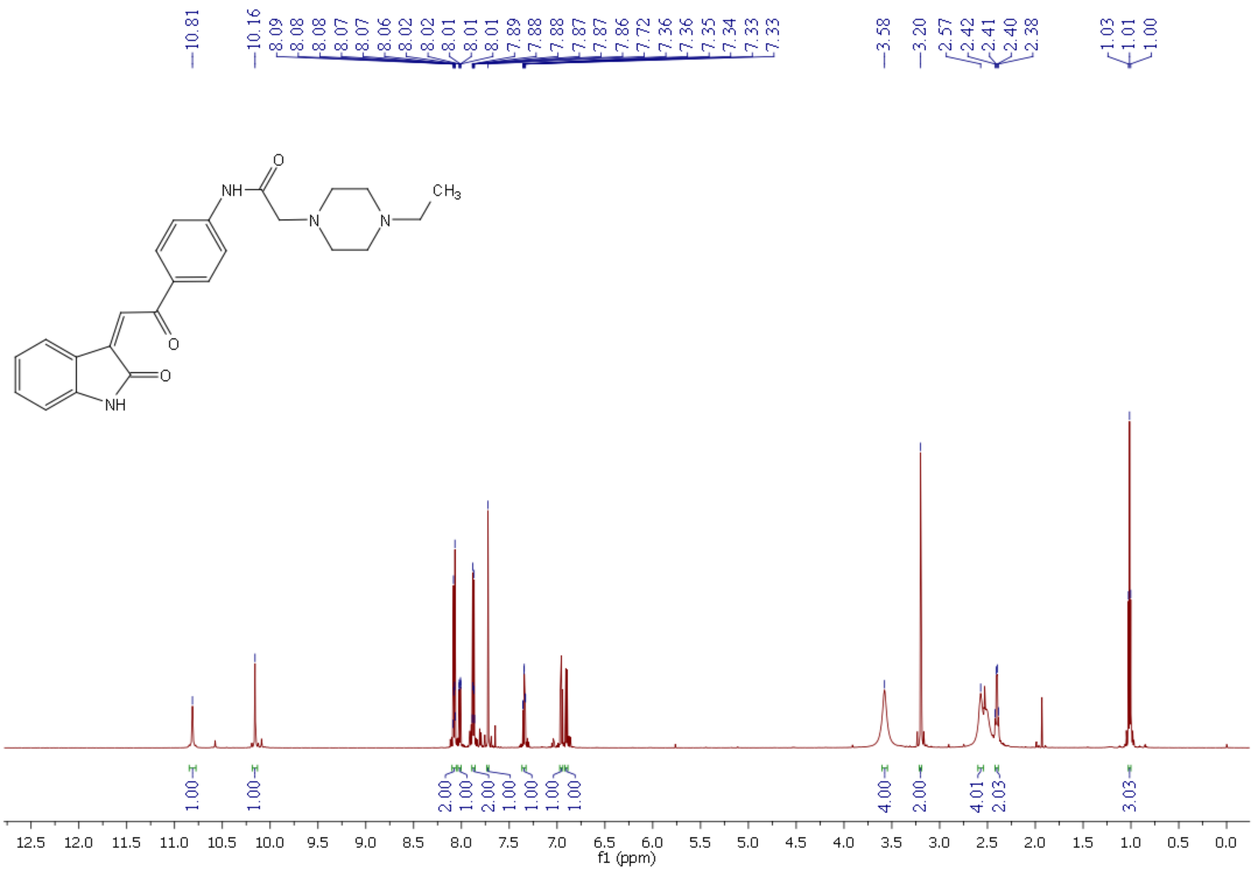

Figure S13: ^{1}H-NMR spectrum of compound **5e** (600 MHz, DMSO/d_6)

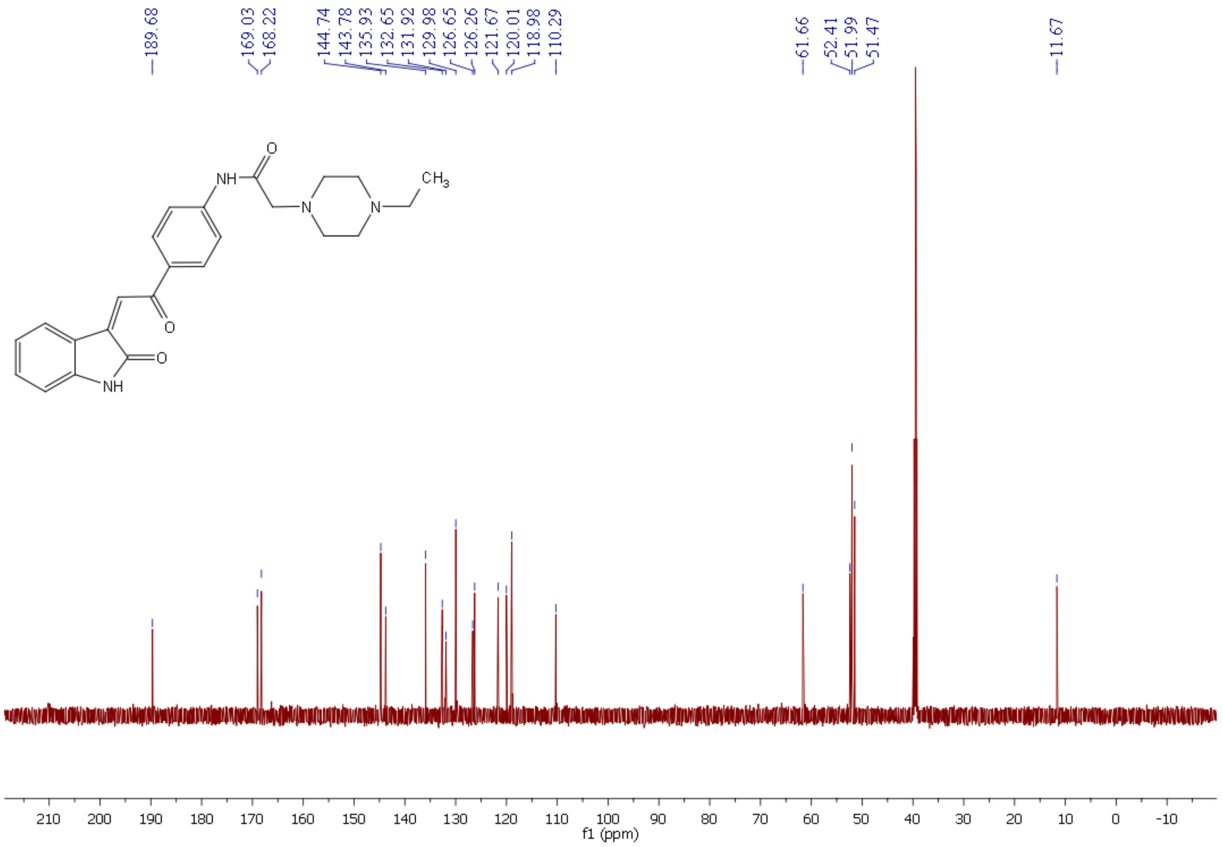

Figure S14: ^{13}C-NMR spectrum of compound **5d** (150 MHz, DMSO/d_6)

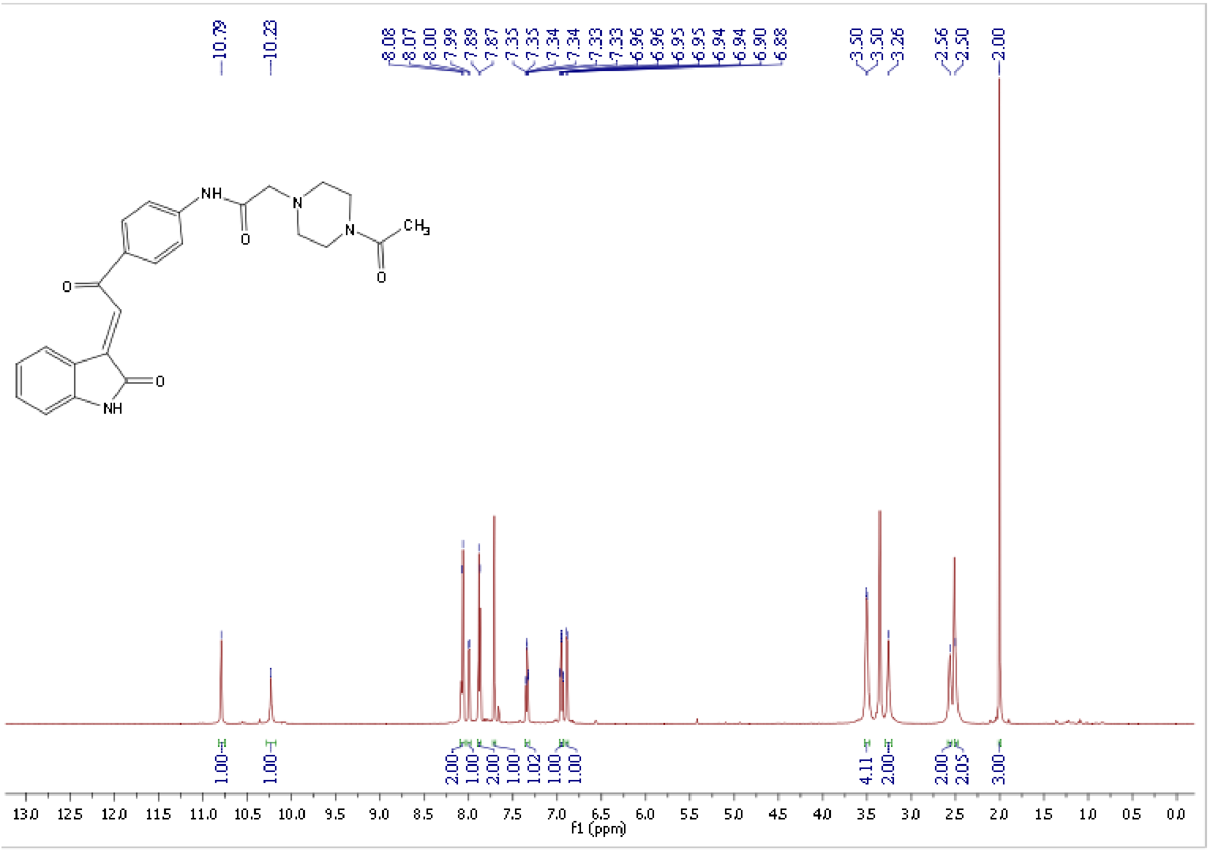

Figure S15: ^{1}H-NMR spectrum of compound **5f** (600 MHz, DMSO/d_6)

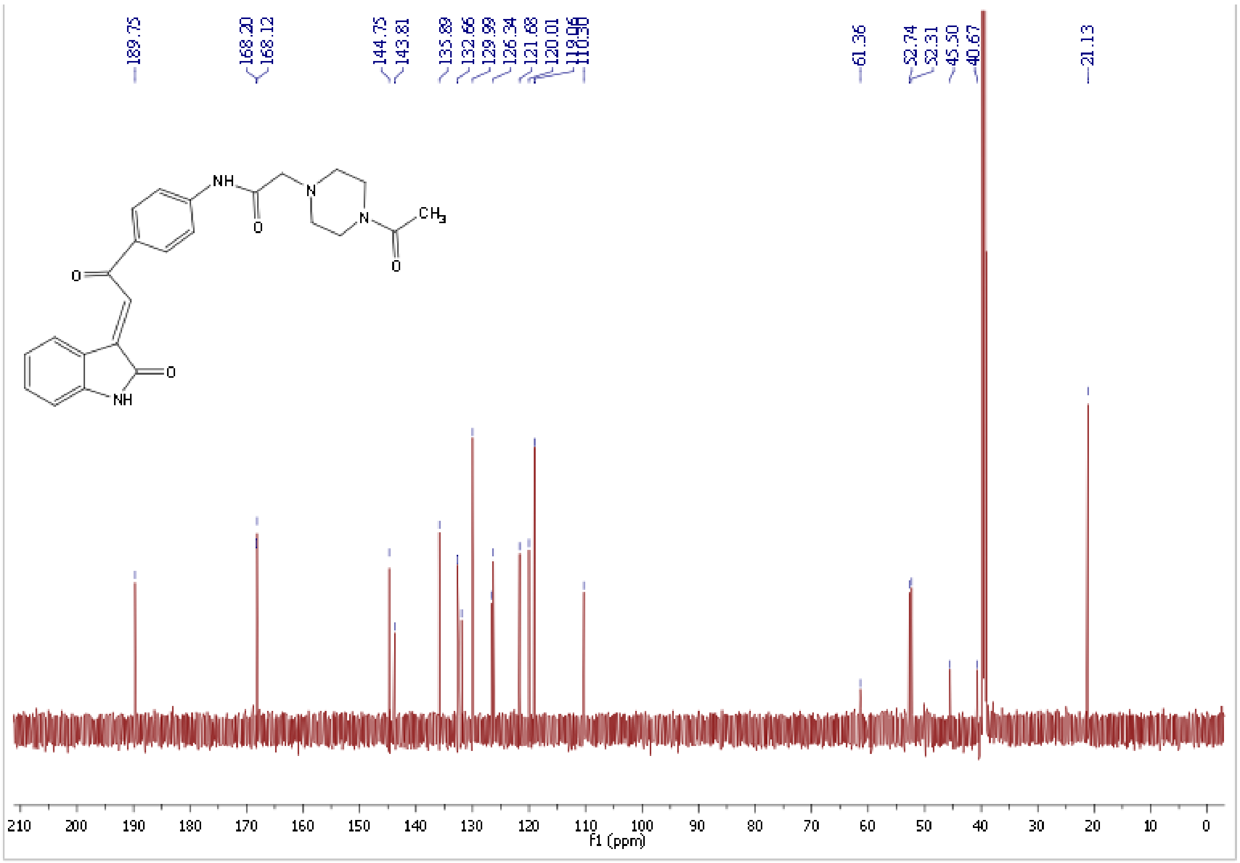

Figure S16: ^{13}C-NMR spectrum of compound **5f** (150 MHz, DMSO/d_6)

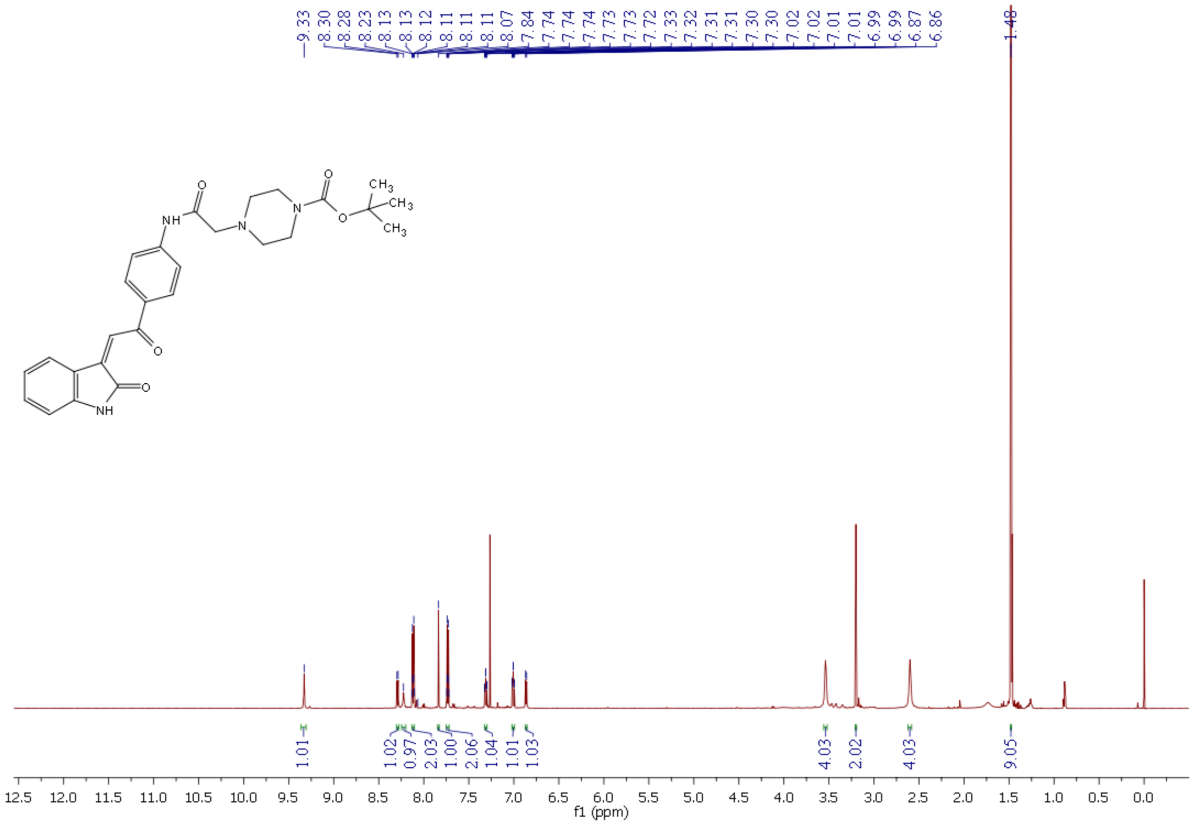

Figure S17: ^{1}H-NMR spectrum of compound **5g** (600 MHz, $CDCl_3$)

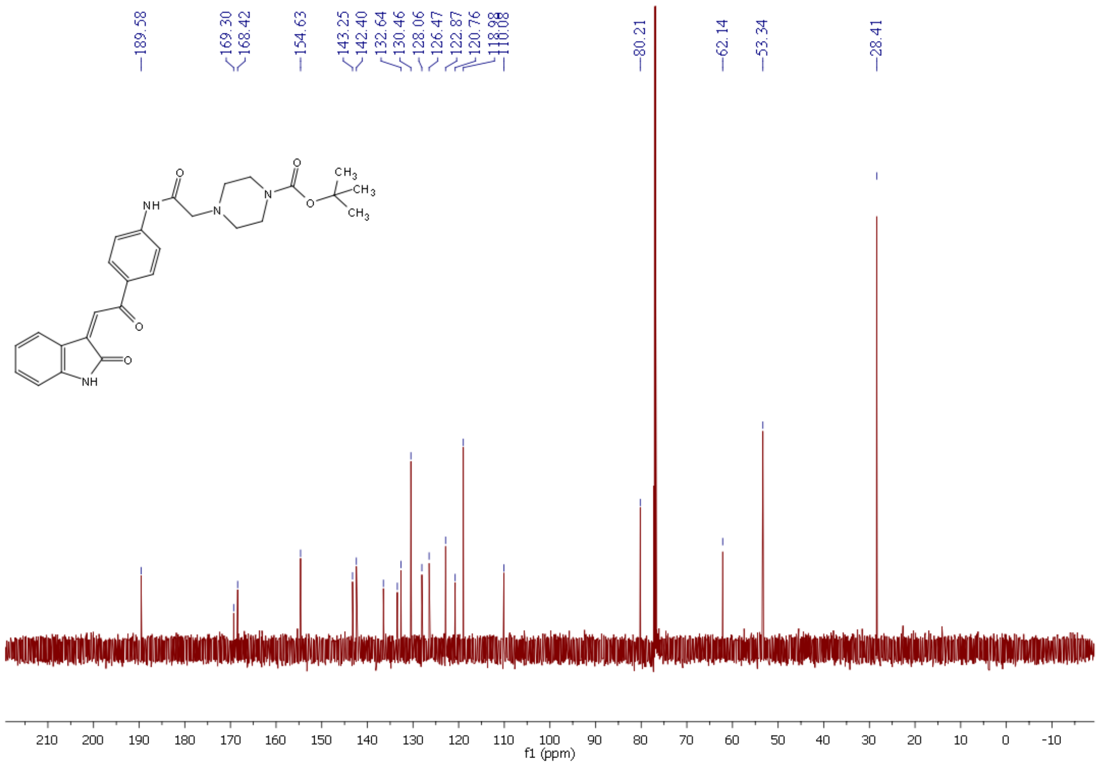

Figure S18: ^{13}C-NMR spectrum of compound **5g** (150 MHz, $CDCl_3$)

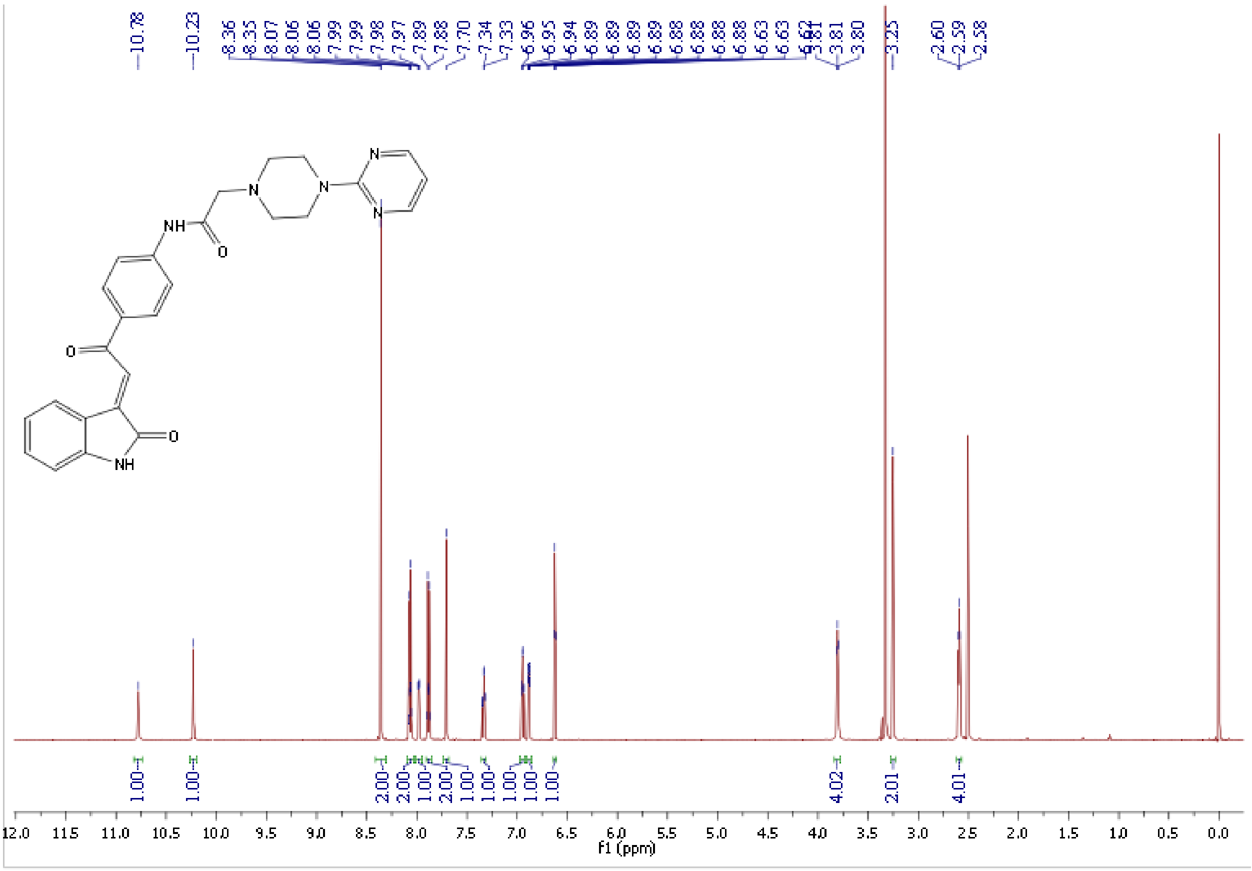

Figure S19: ^{1}H-NMR spectrum of compound **5h** (600 MHz, DMSO/*d*$_6$)

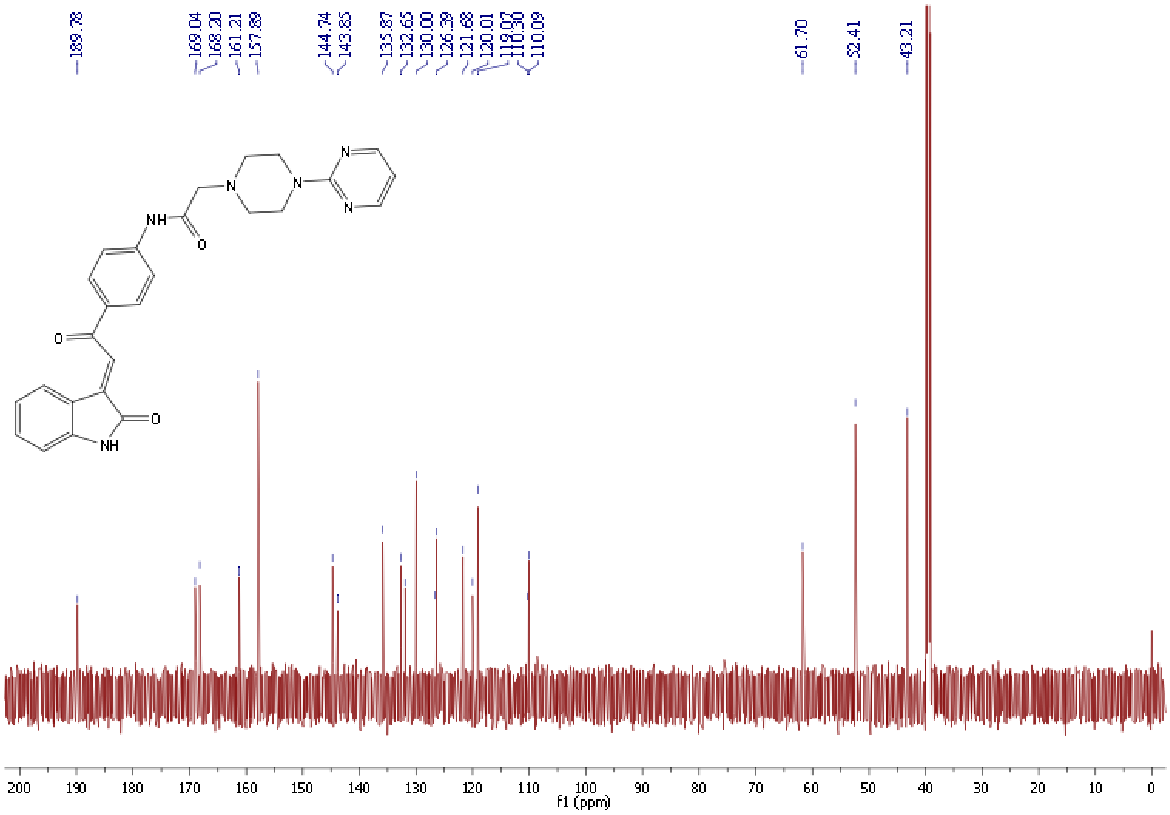

Figure S20: ^{13}C-NMR spectrum of compound **5h** (150 MHz, DMSO/*d*$_6$)

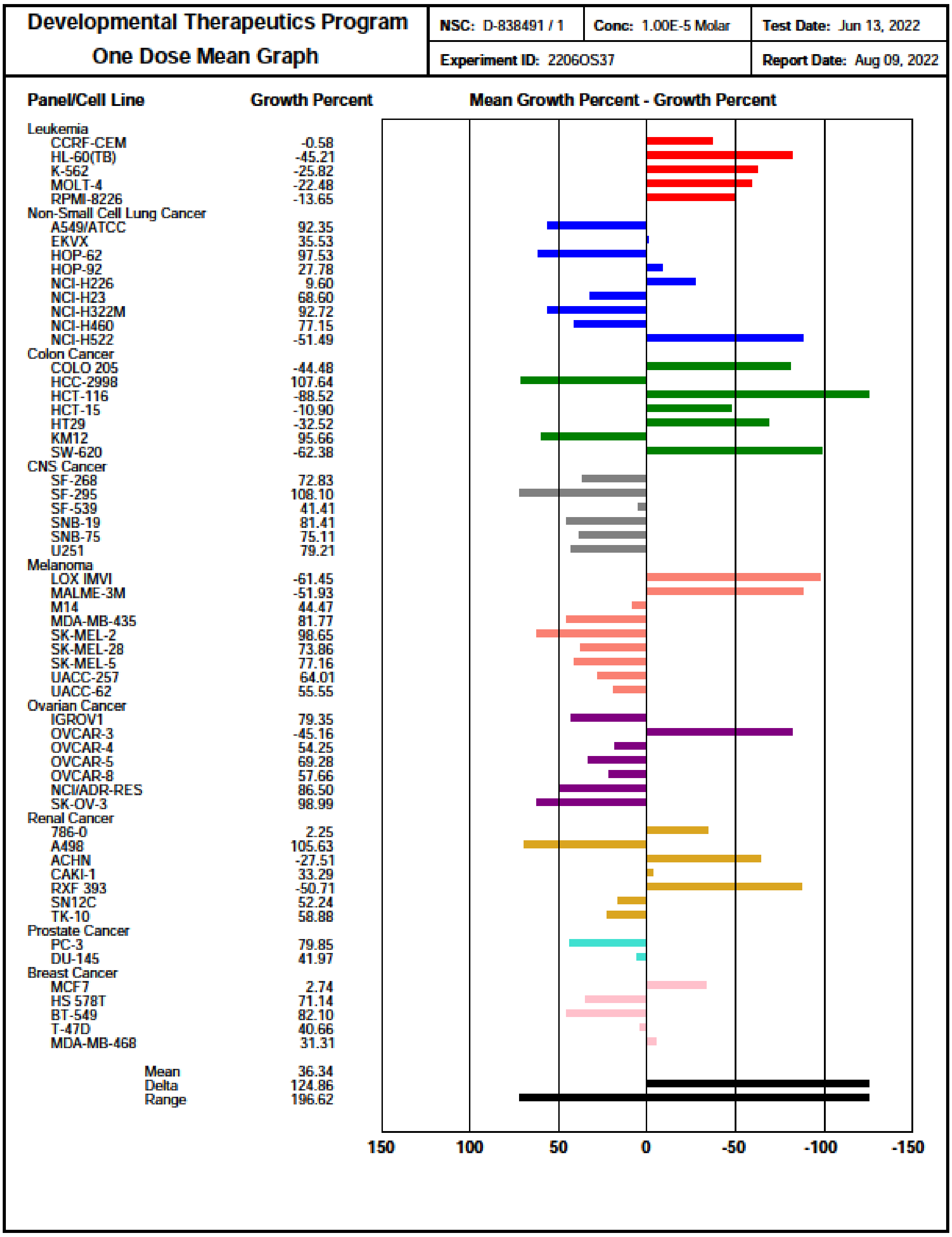

Figure S21. NCI SRB assay mean graph of compound **5a** at 10 μM concentration.

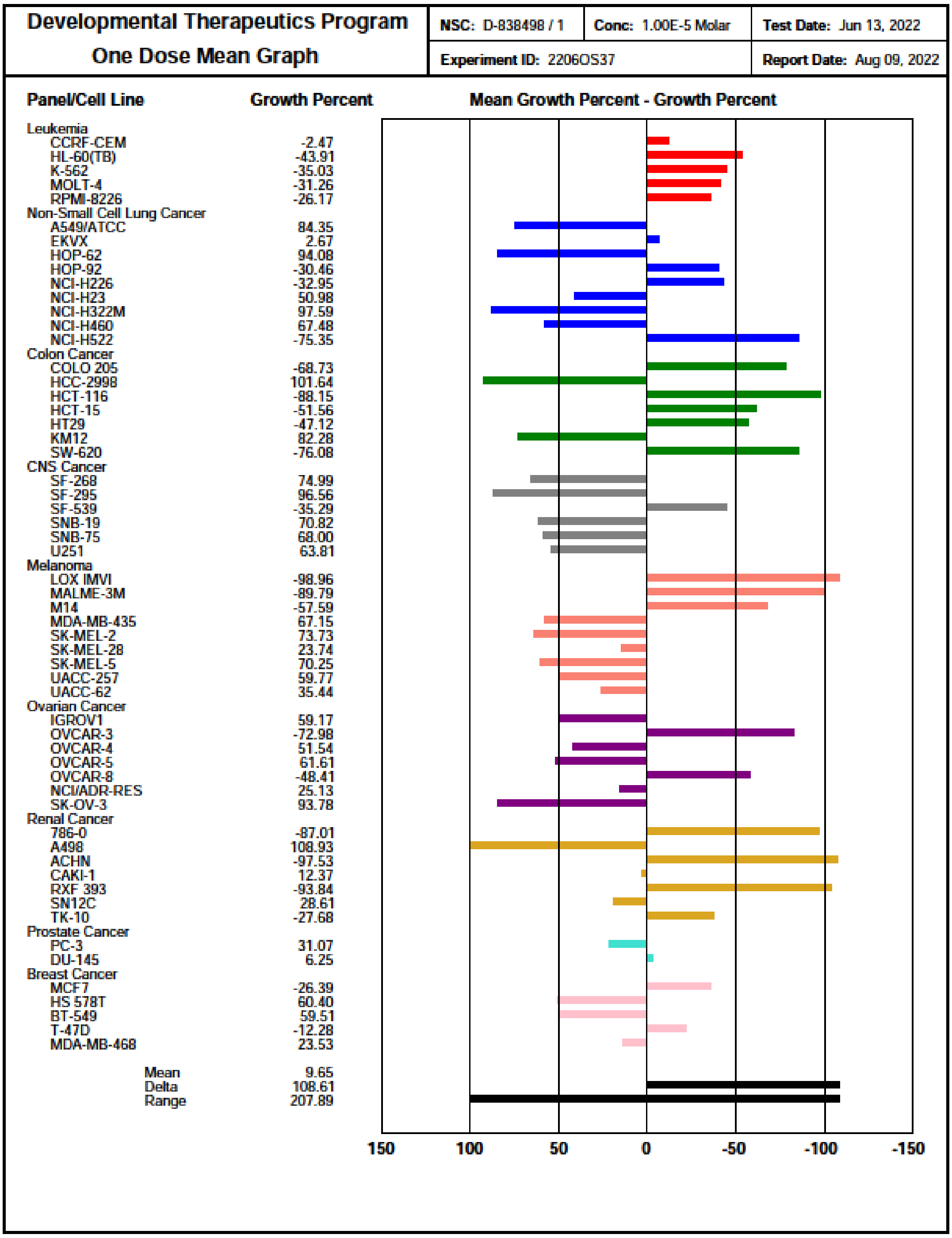

Figure S22. NCI SRB assay mean graph of compound **5b** at 10 µM concentration.

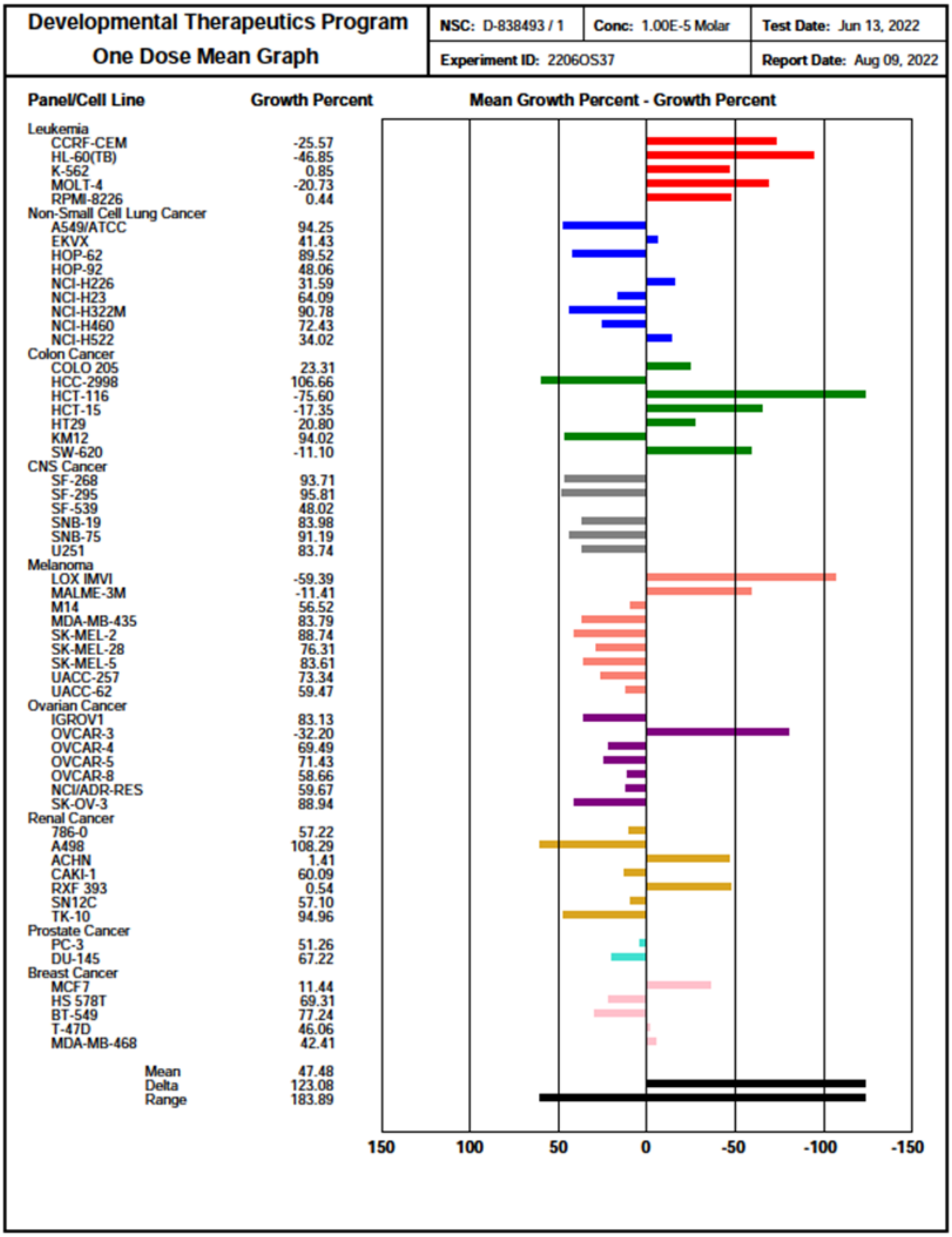

Figure S23. NCI SRB assay mean graph of compound **5c** at 10 μM concentration.

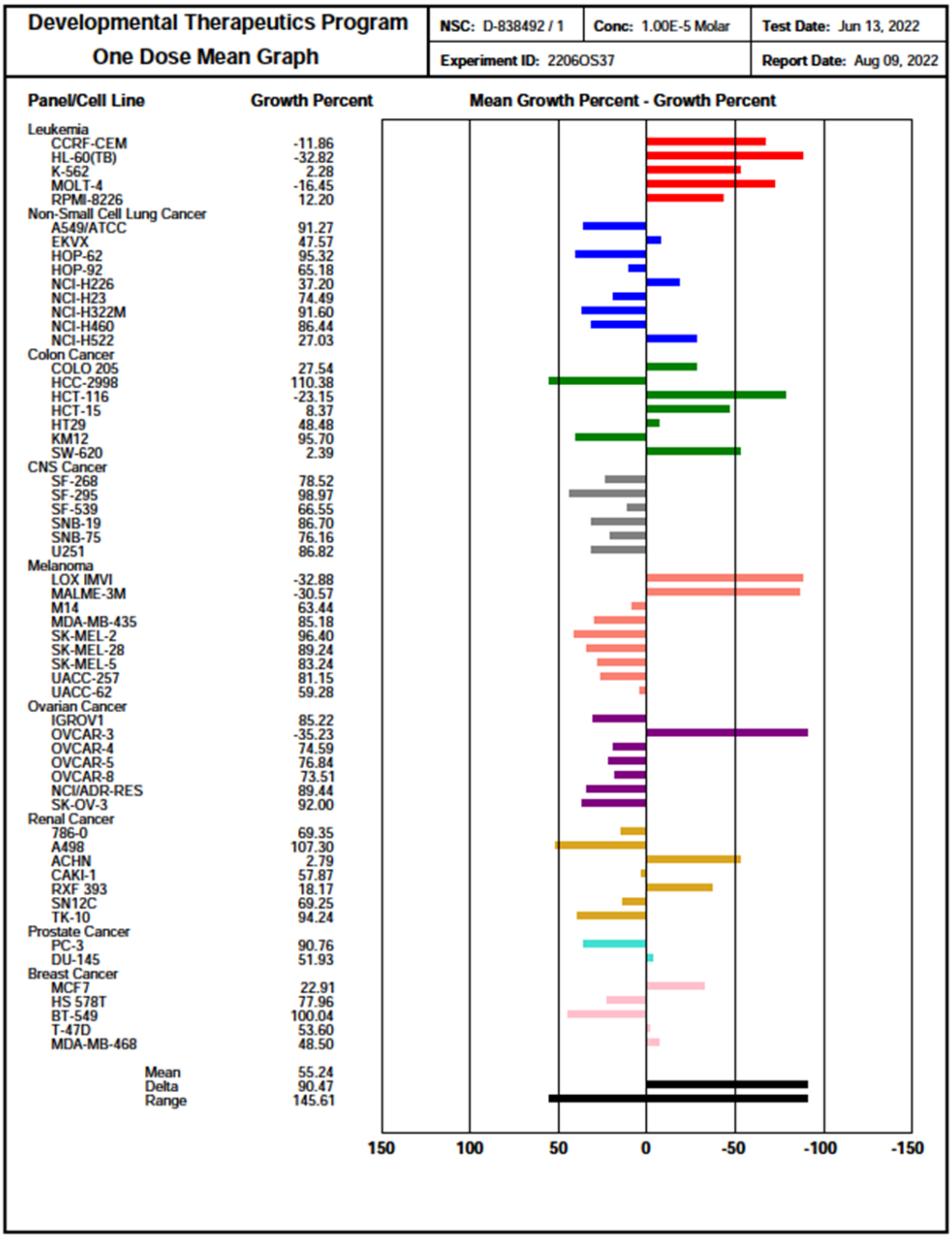

Figure S24. NCI SRB assay mean graph of compound **5d** at 10 μM concentration.

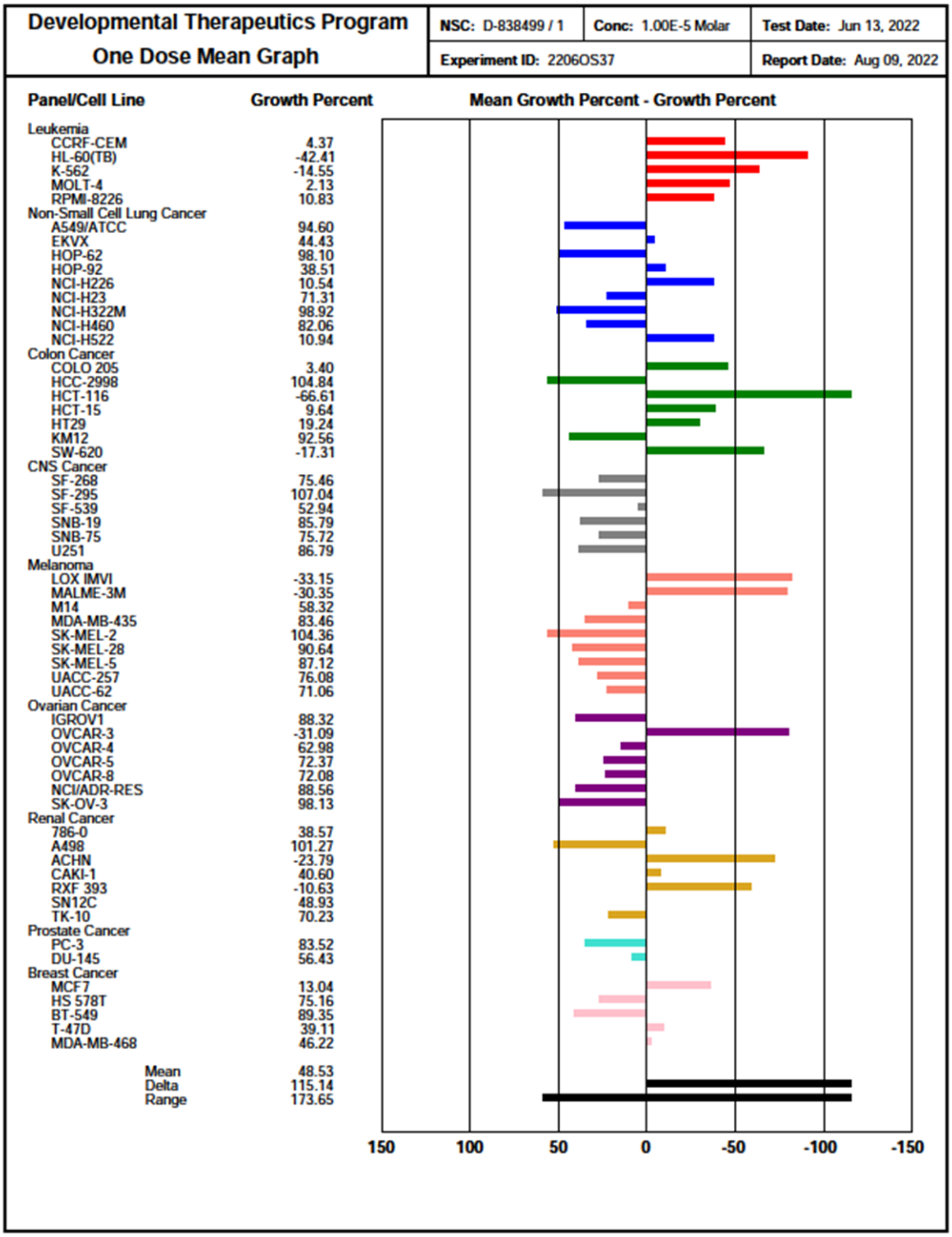

Figure S25. NCI SRB assay mean graph of compound **5e** at 10 μM concentration.

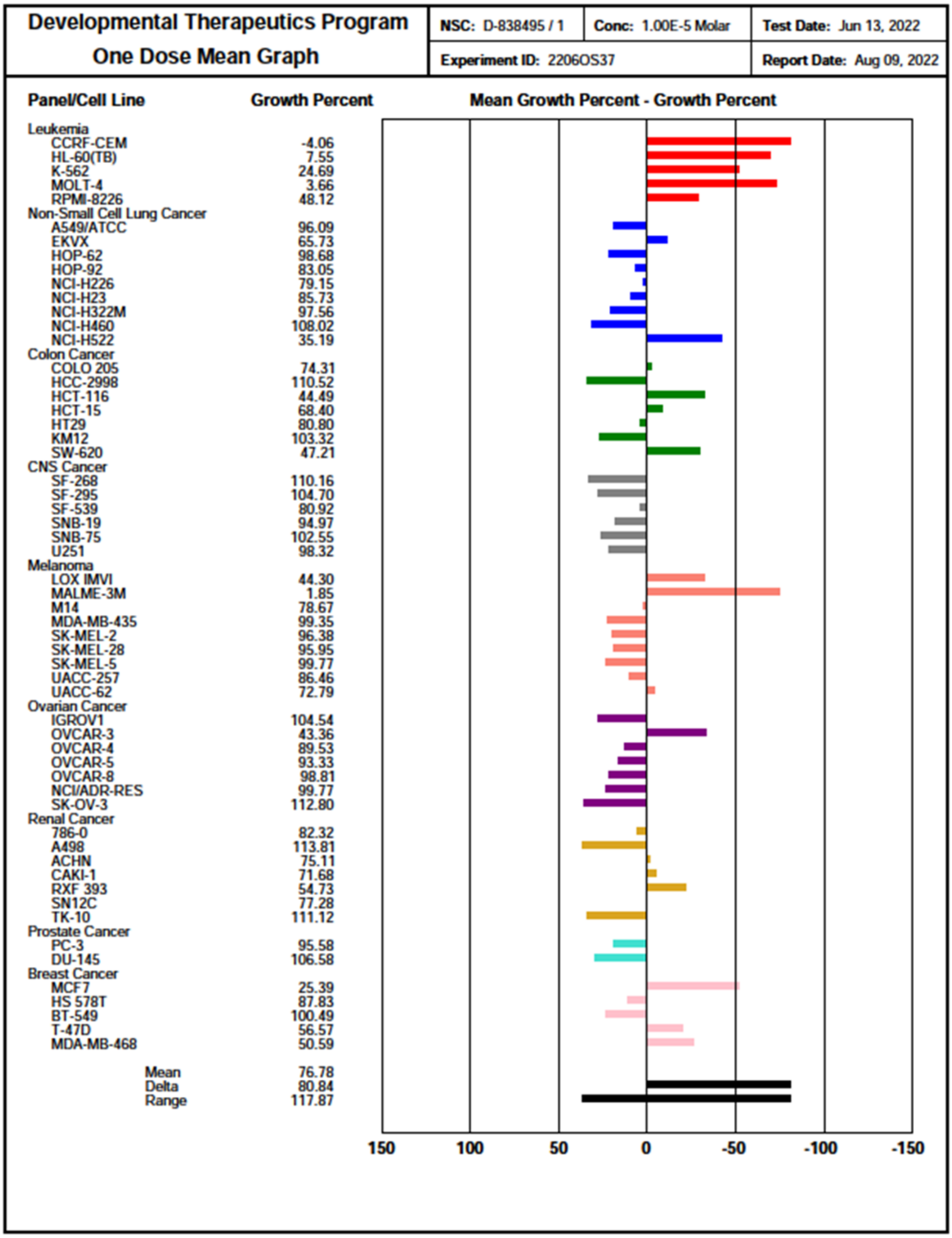

Figure S26. NCI SRB assay mean graph of compound **5f** at 10 μM concentration.

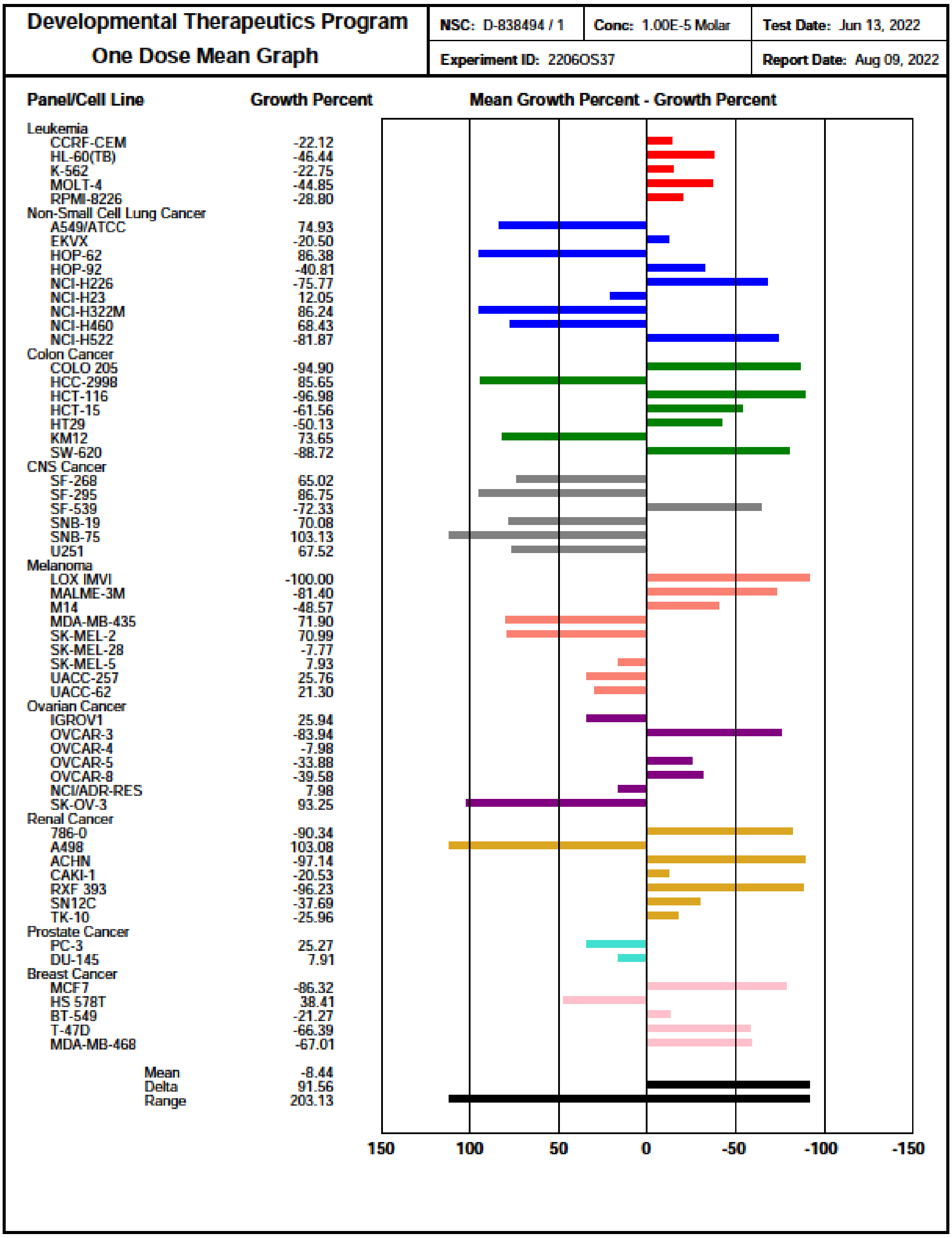

Figure S27. NCI SRB assay mean graph of compound **5g** at 10 μM concentration.

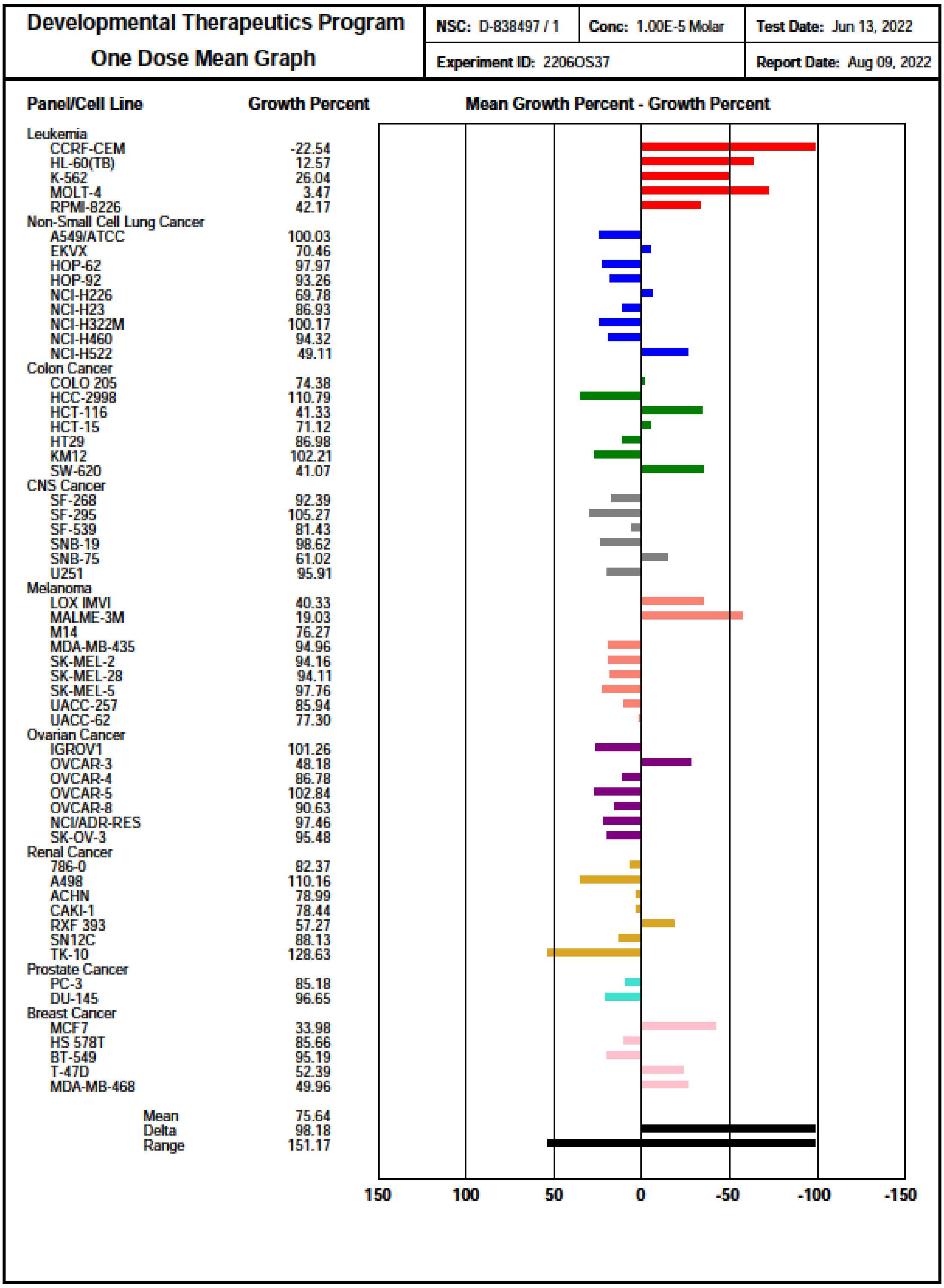

Figure S28. NCI SRB assay mean graph of compound **5h** at 10 μM concentration.
